# Supplementary material for: Man-made microbial resistances in built environments
Source: Nat Commun. 2019 Feb 27;10:968. doi: 10.1038/s41467-019-08864-0 (PMC6393488; doi:10.1038/s41467-019-08864-0)
Supplement: Supplementary file 1 — Supplementary Information [file 41467_2019_8864_MOESM1_ESM.pdf]

Supplementary Information to:

Man-made microbial resistances in built environments

Alexander Mahnert[1*], Christine Moissl-Eichinger[2, 3], Markus Zojer[4], David Bogumil[5], Itzhak Mizrahi[5], Thomas Rattei[4], José Luis Martinez[6] and Gabriele Berg[1, 3]

[1]Institute of Environmental Biotechnology, Graz University of Technology, Graz, Austria
[2]Department of Internal Medicine, Medical University Graz, Austria
[3]BioTechMed Graz, Graz, Austria
[4]Division of Computational Systems Biology, Department of Microbiology and Ecosystem Science, University of Vienna, Austria
[5]Department of Life Sciences, Faculty of Natural Sciences, Ben-Gurion University of the Negev, Israel
[6]Centro Nacional de Biotecnologia, CSIC, Madrid, Spain

*Correspondence:
Alexander Mahnert
Institute of Environmental Biotechnology
Graz University of Technology
Graz, Austria
alexander.mahnert@gmail.com

Supplementary Methods

Detailed description of sampled built environments:

Public buildings as well as public and private houses located in the rural area of a wildlife park (in close touch with nature in Eekholt, Germany) represented the model for a totally unrestricted built environment (UB, Supplementary Fig. S19). The intensive care unit (ICU, Department of Internal Medicine, University hospital, Graz, Austria, Supplementary Fig. S20) in full operation and in a suburban area, act as a model for confined built environments (CB) with special attributes of its occupants [1]. Finally, a highly confined indoor environment (CB) was represented by a cleanroom facility [2] with its adjacent gowning area used for spacecraft assembly by the European Space Agency (ESA) in the urban area of Turin, Italy (Supplementary Fig. S21). Samples throughout these different built environments were obtained from floors to allow a high grade of comparability and sufficient biomass for the applied methods. In addition, samples from ICU floor surfaces were compared with samples from other ICU locations as well, using a targeted approach of the 16S rRNA gene. We also tried to reduce potential technical effects (sampling procedures, DNA extractions, sequencing run effects) as well as distortive environmental effects (sampling season and room locations) by a consistent experimental design and numerous controls throughout our study [3–5]. Such precautions minimize technical differences in our study that could overlay actual biological effects [4].

In particular the following environmental influences should be considered for each sampled category:

The natural unrestricted indoor environment of public and private buildings and houses in a rural area were exposed to the highest degree to the surrounding outdoor environment. A variety of discrete landscapes with respectively associated ecosystems surrounded these structures – including bogs, wetlands, coniferous and deciduous wooded forests with a winding creek in grasslands and pasture including > 700 animals in ~100 different species of insects, fish, reptiles, birds and mammals (www.wildpark-eekholt.de).

The public buildings were located in all these different ecosystems. During the business hours of the wildlife park (from 9 am to 6 pm) these buildings were wide apart for a microbial input via the outdoor air (pollen, seeds, water droplets and other biotic and abiotic particles and compounds)

and various animals (insects, birds, mammals, humans and their pets) with brought along particles associated to soil, foods (snacks), and personal items (e.g. buggies etc.). The rooms of these structures were window ventilated or simply wide apart. Floor surface materials comprised concrete, tiles, polymers and wood. In contrast to the other sampled indoor environments the floors were cleaned only mechanically with a broom and people interacted only marginally with the floor environment, since they primarily observed wildlife, studied boards about the presented ecosystem or simply seek for shelter due to unfavorable weather conditions.

The public houses were so-called ‘A-frame houses’ surrounded by lawns, conifers and deciduous trees adjoining to broad fields of farmland. The houses comprised a recreation room (wooden floor), a kitchen and restrooms (tiled floors). Especially the floor surfaces of the kitchen and the restrooms were treated regularly with natural soaps and were also affected (food preparation, conducting daily hygiene) in a different way compared to the previous category of public buildings. The public houses were used by school classes, who occupied the rooms for a week to receive environmental education. Houses were closed during absence of occupants and window ventilated in the presence of pupils or the cleaning staff.

While a public house is frequented by numerous temporary residents, the private house in contrast is usually inhabited by a family or a reduced number of residents. Likewise, daily behavior and interaction with surfaces is highly personalized. Hence, compared to public houses, this personalized maintenance represents a first step of confinement of an indoor environment. The sampled private house was an old farmyard encircled by a garden with fruit trees and conifers of the adjacent forest. Sampled rooms comprised a kitchen (floor with polymer tiles), a barn mainly used for dining with guests (stone floor) and a conference and locker room (tiled floor) in an adjacent building used as a bird foster station. The house was window ventilated and occupied by the co-founder of the wildlife park - an elderly women and her dog. The resident was supported by a household help and regularly visited by employees of the wildlife park. Floors were cleaned with all-purpose cleaners, the dog received its food in a bowl in the kitchen and the barn was inhabited by swallows during the summertime. Due to the high frequentation of occupants it was uncommon to change shoes before entering the private house (in contrast to many other private households). The same behavior was true for the public built environments described above.

On the contrary to these UB environments, the investigated ICU was located in a suburban area. Compared to public buildings, public and private houses, indoor environments of hospitals are severely different in many aspects: First of all beside staff the majority of people are suffering from

severe infections and other health problems. Therefore these patients might deliver specific microbes like opportunistic pathogens to their surroundings or acquire problematic germs from it. In this ICU critically affected patients suffering from all subspecialties of internal medicine and neurologic defects were treated in 15 beds including an isolation unit for severe immunocompromised patients. All rooms of the ICU were mechanical ventilated by an air conditioning system. Access to the ICU was restricted and controlled by the medical staff. Special garment regulations were applicable in dependence on the grade of interaction with patients or medical devices. A comprehensive maintenance, cleaning and disinfection plan especially characterizes this indoor environment: Waste was removed three times a day and waste containers were disinfected with a surface disinfection cleaner. In the same manner doorknobs and tiles in working height were cleaned twice per day. All-purpose cleaners were used to treat free surfaces and furniture (shelfs, chairs, tables, conduits, window boards, radiators, elevators), a surface disinfectant was used for dirty laundry buckets, handrails, cleaning cars, surface cleaning devices and sanitary glass cleaners and surface disinfectants were used at the bath place for showers and sinks once a day. All other furniture, objects or room installations were cleaned weekly, monthly, per quarterly period, semiannual or annually. Beside restrooms and shower heads all floors were cleaned twice a day or even more often if visible contaminants (blood, feces or other secreted liquids) were observed. If known hospital germs (e.g. *Clostridium difficile*) were detected additional hygiene protocols became effective up to individual isolation of patients. A detailed list of all cleaning products used for disinfecting hands, surfaces, instruments or antisepsis of skin and mucosa are listed in Supplementary Table 4 and Supplementary Table 5 beside other agents to treat known resistant hospital germs and viruses.

An additional level of microbial control is realized in cleanrooms during spacecraft assembly, especially if a celestial body with relevance to extraterrestrial life detection is targeted. According to planetary protection regulations [6] these spacecraft are not only assembled in cleanrooms, but are also subject to regular microbial monitoring, cleaning and removal of surface associated microbes (bioburden control and countermeasures) [7]. Most microbes are intensively reduced not only by the low water and nutrient availability, but especially through intensive HEPA air filtration and the reduction of air-borne particles through air recirculation. Hence, cleanrooms are categorized into different cleanroom classes by the amount and size of particles. Moreover, type and quality of gaseous substances, temperature, humidity, electromagnetics, electrostatics etc. can be controlled. Low emitting surfaces and materials (e.g. floors, doors, door handles, tables, shelves, trolleys) are

mopped with sterile cloths to remove dust, before surfaces are cleaned several times a day with alcohols (e.g. 70% isopropanol), detergents based on hydrogen peroxide (e.g. Klercide-CR), high alkaline cleaning reagents (e.g. Jaminal Plus or Kleenol 30) and sometimes even with vapor-phase hydrogen peroxide or cold plasmas to reduce the bioburden of spacecraft. However, due to the complexity of working processes during spacecraft assembly, engineers still have to interact with spacecraft components and are therefore a main source of microbial dispersal. As a counter measurement strict gowning protocols with special cleanroom garment have to be followed in repetitive steps and sluices and air showers define transitions through different cleanroom classes and rooms. Finally strict protocols (e.g. slow body movements) regulate the interaction of engineers and staff with the cleanroom environment, spacecraft components and the assembled spacecraft itself to minimize the dispersal of human associated microbes into the cleanroom.

All sampled built environments could be characterized by distinct environmental features such as the geographic location, microclimate, architecture, room maintenance and usage (Table 1). Whereas samples from unrestricted environments were located at low elevations in northern Germany, samples from confined built environments originated from higher altitudes in south-east Austria and north-west Italy. Concerning respective microclimates, unrestricted environments of public buildings and public as well as private houses were specified by higher relative air humidity and lower temperatures, compared to controlled environments of the ICU, and the cleanroom facility with its gowning areas. The ICU highlighted most opposed climatic conditions: with the highest room temperature (24°C) and the lowest relative air humidity (32%). Further discriminating attributes were represented by architectural features of sampled built environments. Here, cleanrooms represented very large indoor room surfaces compared to small spaced infrastructure in the case of gowning areas, rooms in the ICU, public buildings and public and private houses. The smallest room volumes were represented in private houses. Likewise, more controlled built environments harbored only a few defined synthetic materials on floors, compared to a higher variety of floor materials including materials of natural sources like wood and stone in unrestricted built environments. Moreover, occupancy and intensity of cleaning were obviously different between public buildings, public and private houses, the ICU and the cleanroom facility with its gowning areas. Hence, a higher grade of environmental confinement resulted in more elaborative maintenance and cleaning procedures, but a lower grade of occupancy.

Sample processing:

Alpha Wipes® (TX1009, VWR International GmbH, Vienna, Austria) were extracted in 40 mL 0.9% sterile DNA-free sodium chloride solution by vortexing for 10 seconds and sonication at 40 kHz for 2 min in an ultrasonic bath (Transonic Digitals, Elma, United States). Extracted sampling liquids were concentrated by repeated centrifugation cycles at 3220 g and 4°C for 5 min using UV sterilized Amicon filter tubes (Amicon Ultra-15 Centrifugal Filter Units, Merck, Germany) to a final volume of 500 µl.

PMA treatment of samples:

An aliquot of selected biological samples and spot tests of used reagents were treated with propidium monoazide (PMA) according to manufacturer instructions (GenIUL, S.L., Terassa, Spain). Samples were treated with a final concentration of 50 µM PMA for 30 min. in the dark. Afterwards treated and non-treated samples were exposed in parallel to the PhAST blue-Photo activation system for tubes (GenIUL, S.L., Terrassa, Spain) for 15 min.

DNA extraction:

The DNA extraction procedure included mechanical, thermal and chemical lysis of the cells. All cell suspensions were homogenized with a FastPrep-24 Classic Instrument (MP Biomedicals, United States) in two cycles for 30 seconds at 6.5 m/s. Homogenized samples were then added to an equal volume of freshly prepared XS buffer (2x). A ~20 ml stock solution of the XS buffer (2x) contained: 4 ml of 1M Tris/HCl (pH 7.4); 4.56 ml of 7M ammonium acetate; 3.2 ml of 250 mM ethylene diamine tetraacetic acid; 4 ml of 10% (w/v) sodium dodecyl sulfate; 0.4 g of potassium ethyl xanthogenate; and 4.99 ml of PCR-grade water. The XS buffer was incubated at 65°C for 15 min. to dissolve the xanthogenate completely. The homogenized cell suspension in the XS buffer was incubated at 65°C for 2 hours and gently mixed by inverting the tube every 30 min. After the incubation period, the tubes were vortexed for 10 seconds and placed on ice for 10 min, followed by centrifugation at 100 g and 4°C, for 5 min. The supernatant was transferred into a PhaseLock Gel tube (Eppendorf, Hamburg, Germany), and an equal volume of phenol: chloroform:isoamyl alcohol (25:24:1) was added. Sample suspensions were mixed and centrifuged at 2000 g and 15°C for 5 min. The upper aqueous layer was transferred into a new tube. For DNA precipitation, the same volume of cold 100% isopropanol and 1/10 volume of 4M ammonium acetate was added. Suspensions were gently mixed and incubated at -20°C overnight. On the next day the precipitated

DNA was centrifuged at 13600 g and 4°C for 30 min. Invisible DNA pellets were washed with 1 ml of ice-cold 70% ethanol and centrifuged again at 13600 g and 4°C for 30 min. DNA pellets were dried completely in a clean bench and finally dissolved in 50 µl of PCR-grade water.

qPCR:

Quantitative molecular measures were based on the primer pair 515F – 927R (10µM each, Supplementary Table 6). For the qPCR run DNA templates were amplified in 40 cycles with denaturation at 95°C for 20 sec., annealing at 54°C for 15 sec. and elongation at 72°C for 30 sec. on a Rotor-Gene™ 6000 real – time rotary analyzer (Corbett Research, Sydney, Australia). A melt curve from 72°C to 95°C served as a quality control for the amplified products. One qPCR reaction mix was constituted as follows: 1.06 µl PCR grade water, 3.5 µl KAPA Plant PCR buffer (KAPA3G Plant PCR Kit, Peqlab, VWR International GmbH, Erlangen, Germany), 0.42 µl forward and reverse primers, 0.056 µl of KAPA3G Plant DNA-polymerase (2.5 u per µl), 0.78 µl of SYBR® Green (4x concentrate, Invitrogen™, Eugene, OR, USA), and 0.8 µl of the extracted DNA template. qPCR runs with a mean reaction efficiency of 0.84 and mean standard curve R^2 values of 0.99 (16S rRNA gene product of *Bacillus subtilus* B2G) were evaluated in triplicate and counts in negative controls were subtracted from all other samples in their respective qPCR runs.

Plasmidome assembly methods:

The plasmidome of each sample was assembled using the recycler pipeline [8]. Paired end reads were assembled de novo into contigs and the assembly graph, connecting these contigs via shared kmers, was obtained using SPAdes [9]. In order to calculate the read coverage for each contig from the assembly, paired end reads were aligned to the contigs using the Burrows-Wheeler aligner (BWA) [10]. Contigs were subsequently assembled into cycles defined by connectivity in the assembly graph form the de novo assembly and uniform coverage distribution around the circular combination of contigs, using the recycler Software [8]. Plasmid encoded open reading frames (ORFs) were then predicted upon these cycles using Metagenemark [11]. Annaotation of ORFs was performed by blast+ [12] searches against the KEGG FTP release 2017-03-27 database [13], the Uniref90 release 2016_10 database [14] and the CARD 1.1 database [15].

Synteny analysis:

The genomic context of resistance genes (20 kb, 10 kb up -and downstream), pan-genomes, and virulence was analyzed in MaGe [16]. Annotation of virulence factors was based on VFDB [17] and VirulenceFinder [18]. Pan-genomes were calculated with the SiLiX software [19]. IntegronFinder [20] was applied to detect integron clusters. Regions of genome plasticity (RGP) were based on PkGDB organisms and NCBI RefSeq organisms showing highest similarity with the query genome by using RGP Finder and the tools AlienHunter [21] and SIGI-HMM [22].

16S rRNA gene amplicons:

Four individual PCR reactions à 50 µl (17.6 µl PCR grade water, 25 µl KAPA Plant PCR buffer, 0.4 µl KAPA3G Plant DNA-polymerase (2.5 u per µl), 3 µl forward and reverse barcoded primers 515f and 806r (Supplementary Table 6) and 1 µl extracted DNA template) were pooled after successful amplification (40 cycles of 95°C for 30 sec. denaturation, 60°C for 15 sec. annealing and elongation at 72°C for 12 sec.) on a TECHNE TC-PLUS gradient thermocycler (Bibby Scientific Ltd, Stone, UK) and validated by gel electrophoresis. Pooled PCR products were cleaned with the Wizard SV Gel and PCR Clean-Up System kit (Promega, Madison, WI, USA) and measured on the NanoDrop instrument (Thermo Scientific, Wilmington, DE, USA). Equimolar concentrations of PCR amplicons were pooled and sent for sequencing at Eurofins Genomics GmbH, Ebersberg, Germany. The pool was additionally purified by gel extraction and quantified before the sequencing library was prepared by adaptor ligation, PCR amplification according to PCR product insert size and a final library purification and quality control. Sequencing of amplicon samples was performed on an Illumina MiSeq instrument with v3 chemistry and the 2 x 300 bp paired-end read module. Amplicon sequences of the ICU were generated and sequenced as described in [1].

16S rRNA gene amplicon data analysis:

Sequences of 16S rRNA gene amplicons of all indoor environments were analyzed in QIIME 1.9. and QIIME 2 (versions 2017.10 to 2018.11) [23,24], according to [25,26] and developer provided tutorials. Forward and reverse reads were joined with a minimum overlap of 100 bp and a maximum allowed difference of 3%. Barcodes were extracted for demultiplexing and quality filtering of reads with default parameters. Reads in controls were removed by blast (100% alignment cutoff). 454 reads

of ICU samples were denoised with mothur [27], demultiplexed and quality filtered before they were concatenated with the filtered Illumina reads. All sequences were then trimmed to the same 16S rRNA gene regions and lengths (FASTX-toolkit by Assaf Gordon, accessed on Galaxy, galaxyproject.org). Reads were additional quality filtered to remove primer sequences before chimeric sequences were removed with USEARCH [28] giving both Silva 119 and the Greengenes 13_8 release as a reference. OTUs were picked with USEARCH to the same reference and every sequence not present was clustered denovo at 97% similarity level. For phylogenetic based metrics and measures a phylogenetic tree was calculated. The OTU table was filtered for singletons, doubletons, assigned reads to chloroplasts or mitochondria and sorted for following read normalizations in alpha and beta diversity analysis and statistics. Since jackknife supported bootstrapped trees showed higher confidence for weighted than unweighted unifrac measures, weighted metrics were preferred throughout the analysis as also recommended by [3]. OTUs present in the reference were examined for their functional potential with PICRUSt [29]. Potential microbial phenotypes were predicted with BugBase [30]. OTU networks were based on the assigned taxonomy, calculated in QIIME and visualized in Cytoscape [31] as described earlier [32]. Sample metadata predictions were based on random Forest classification and regression [33]. Associations between microbial composition and environmental variables were assessed by bioenv analysis [34] and verified by multivariate linear models using MaAsLin [35], balances in gneiss [36] and linear mixed effects [37].

Statistics on sequencing reads:
3.0 - 6.7 x 10^7 sequences per sample could be obtained via shotgun Illumina HiSeq sequencing. After filtering, a range of 7 x 10^6 to 2.5 x 10^7 quality sequences (phred score ≥ q36) could be retained for following assemblies and binning attempts (Supplementary Table 7). Assemblies on Ray Meta resulted in a satisfying amount of small (≥ 100 nt) and large (≥ 500 nt) contigs and scaffolds (range of N50 values: 142 to 2510, Supplementary Table 8). From these contigs and scaffolds 125 bins (8 to 20 bins per sample) could be generated. Most bins were obtained from the private house, while only a few genomes could be binned from the ICU dataset (Supplementary Table 2). In general, most markers (1389) and marker sets (369) could be binned into 39 genomes assigned to the genus *Pseudomonas* with a completeness of 93.47%, contamination of 2.51%, and heterogeneity of strains of 26.67%.

Supportive 16S rRNA gene amplicon analysis for higher resolutions of respective built environment locations resulted in 225 to 37,831 sequences per sample from a total of 837,216 quality sequences and 10,814 assigned OTUs (Supplementary Table 10, Supplementary Table 11 and Supplementary Table 12).

Verification of bioenv results:

Associations of the microbiome with microclimate or location specific variables could not be further distinguished. MaAsLin was able to define specific taxa (distinct sets, only 6 of 82 were overlapping) for microclimate and location specific variables (e.g. microclimate: *Bauldia, Gaiella* and *Intrasporangium*; location: *Commensalibacter*, *Chlorocromatium*; both: *Iamia*, *Rubrobacter*). However, regression models using balances in gneiss showed that microclimate and location dependent variables contributed to similar proportions (~2%) to the total explained community variation (~70%). Moreover over-fitting of the model could not be ruled out (in 4 out of 6 cross-validations the prediction accuracy was higher than the within model error). Finally, linear mixed effect models were used to test if microbial composition changed over microclimate or location in response to confinement and architecture (room size). This analysis showed that microbial composition was not significantly impacted by these selected variables. Hence, we concluded that environmental variables of the microclimate and the location were confounded in our sample design and were not appropriate to tell if the microclimate or the location has a bigger impact on the microbial composition.

Supplementary Figures

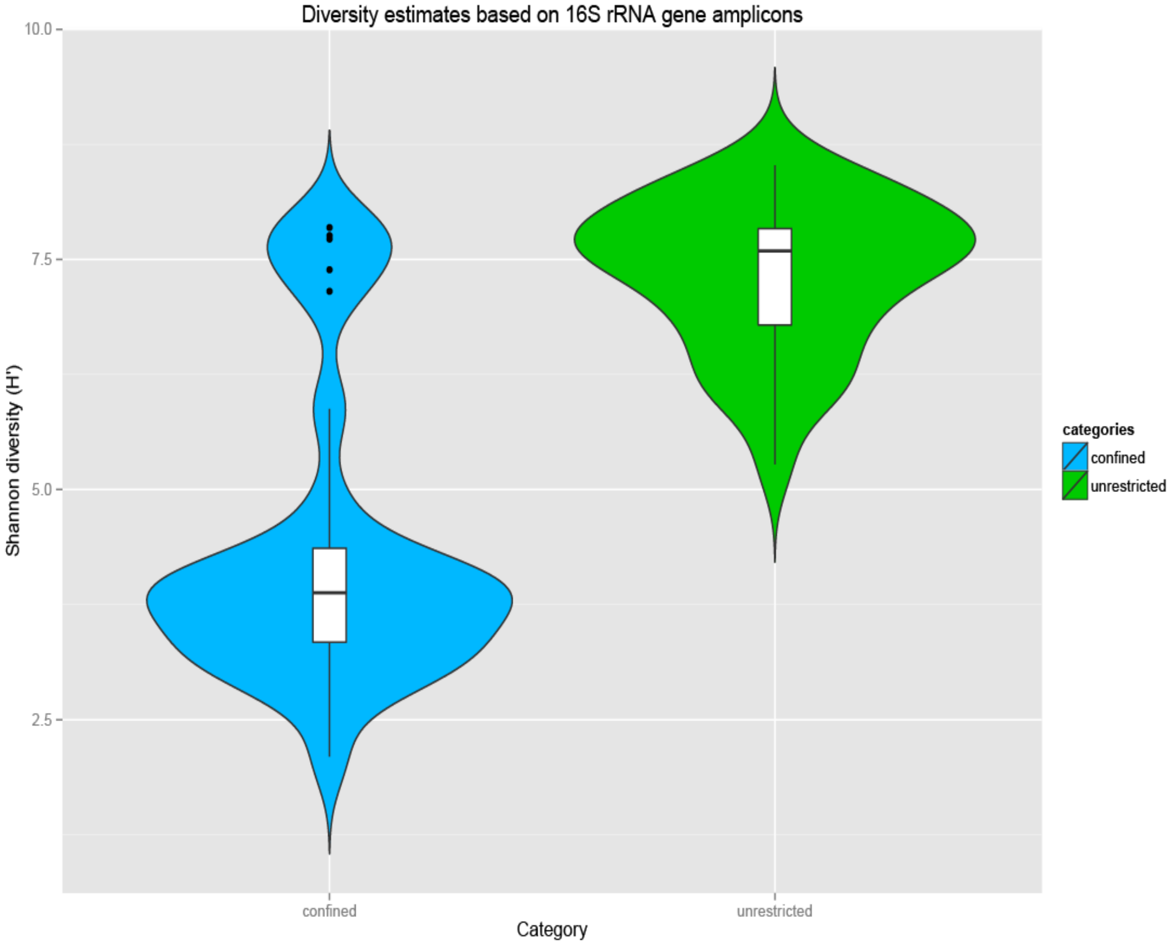

Supplementary Figure 1: **Diversity estimates**

Diversity estimates of confined and unrestricted built environments based on 16S rRNA gene amplicon analysis.

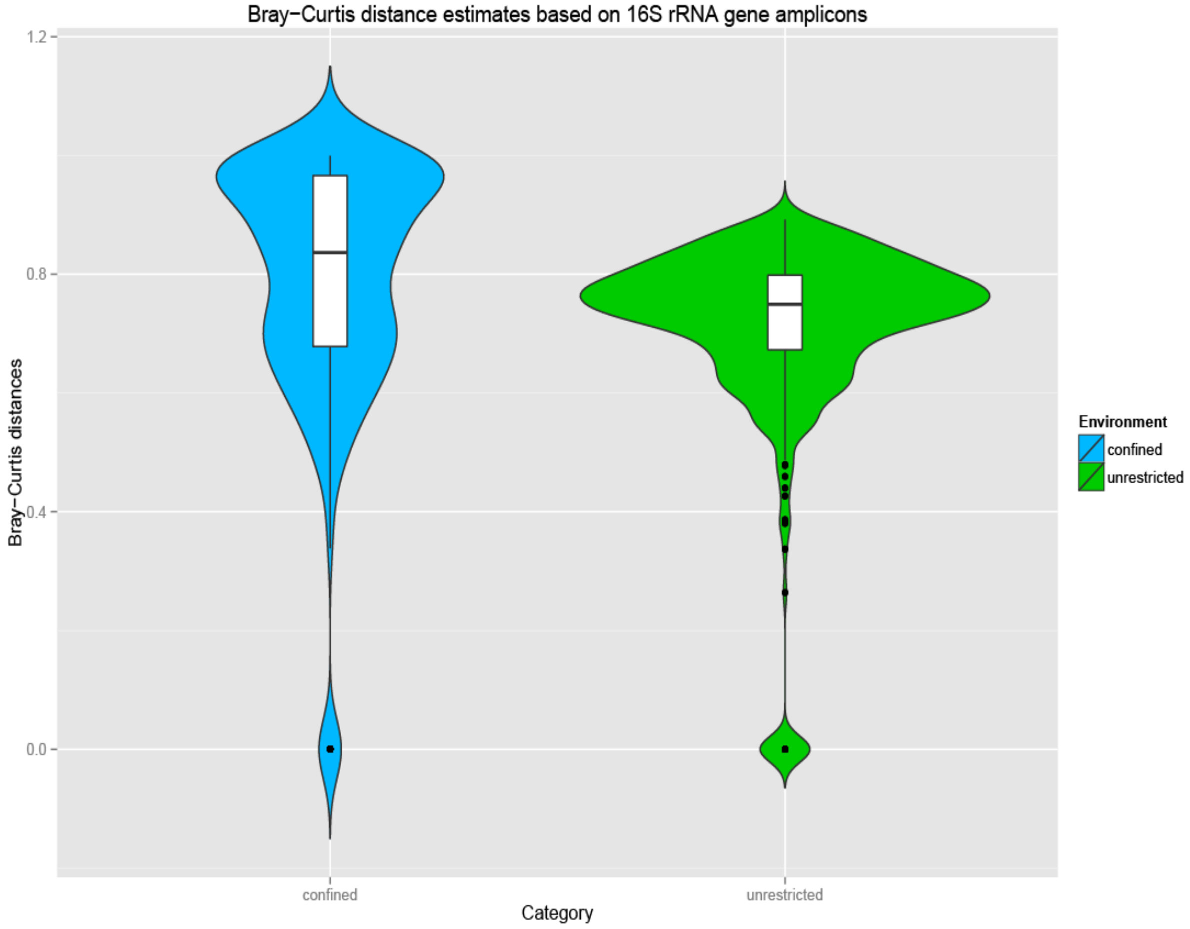

Supplementary Figure 2: **Distance estimates**

Bray-Curtis distance estimates of confined and unrestricted built environments based on 16S rRNA gene amplicon analysis.

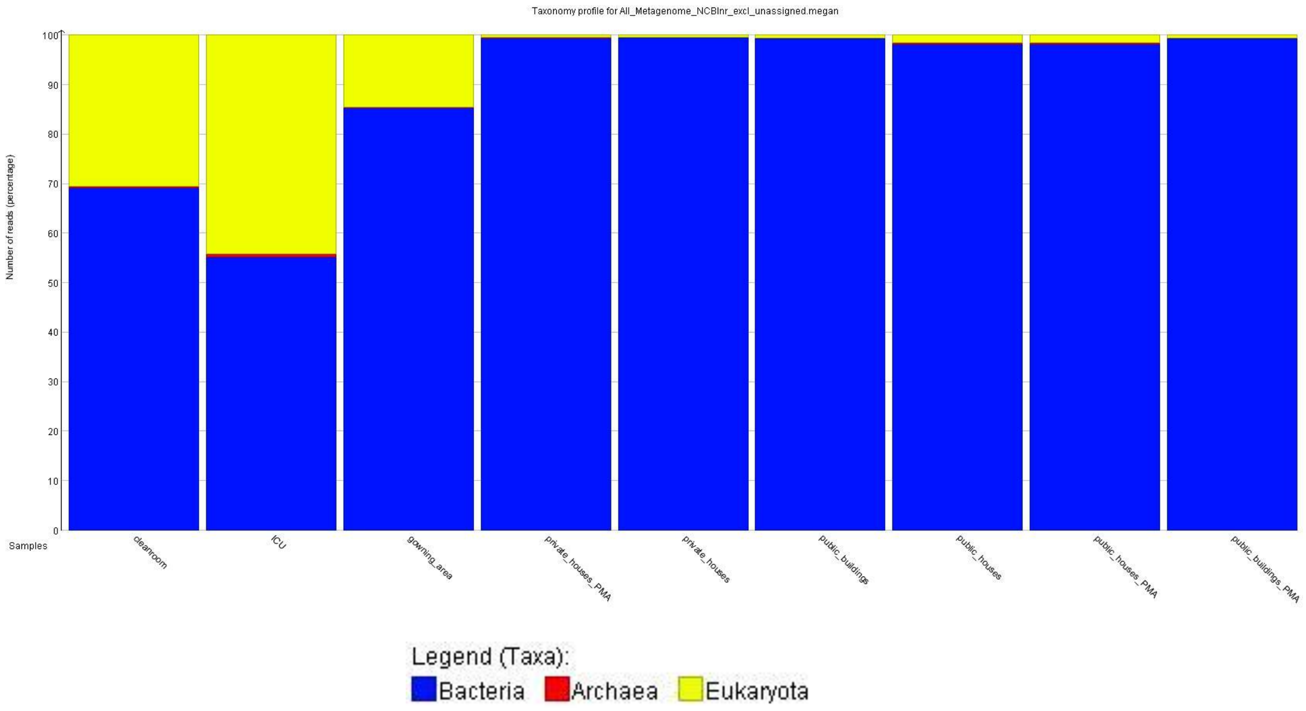

Supplementary Figure 3: **Domain profile**

Single reads BLASTx (rapsearch and diamond) vs. NCBI nr. superkingdom level (derived from MEGAN, excluding unassigned reads, normalized data set).

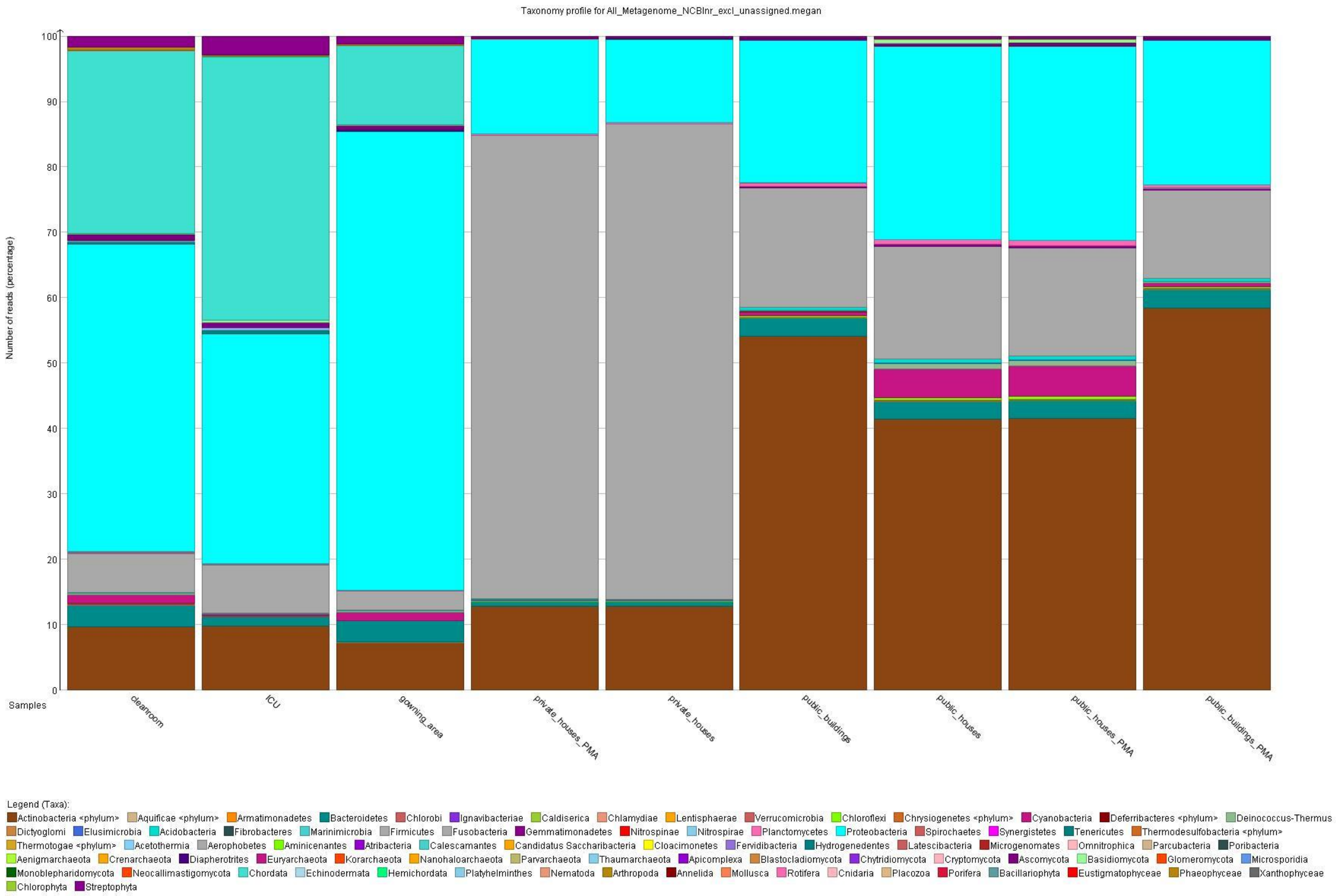

Supplementary Figure 4: **Phyla profile**

Single reads BLASTx (rapsearch and diamond) vs. NCBI nr. phylum level (derived from MEGAN, excluding unassigned reads, normalized data set).

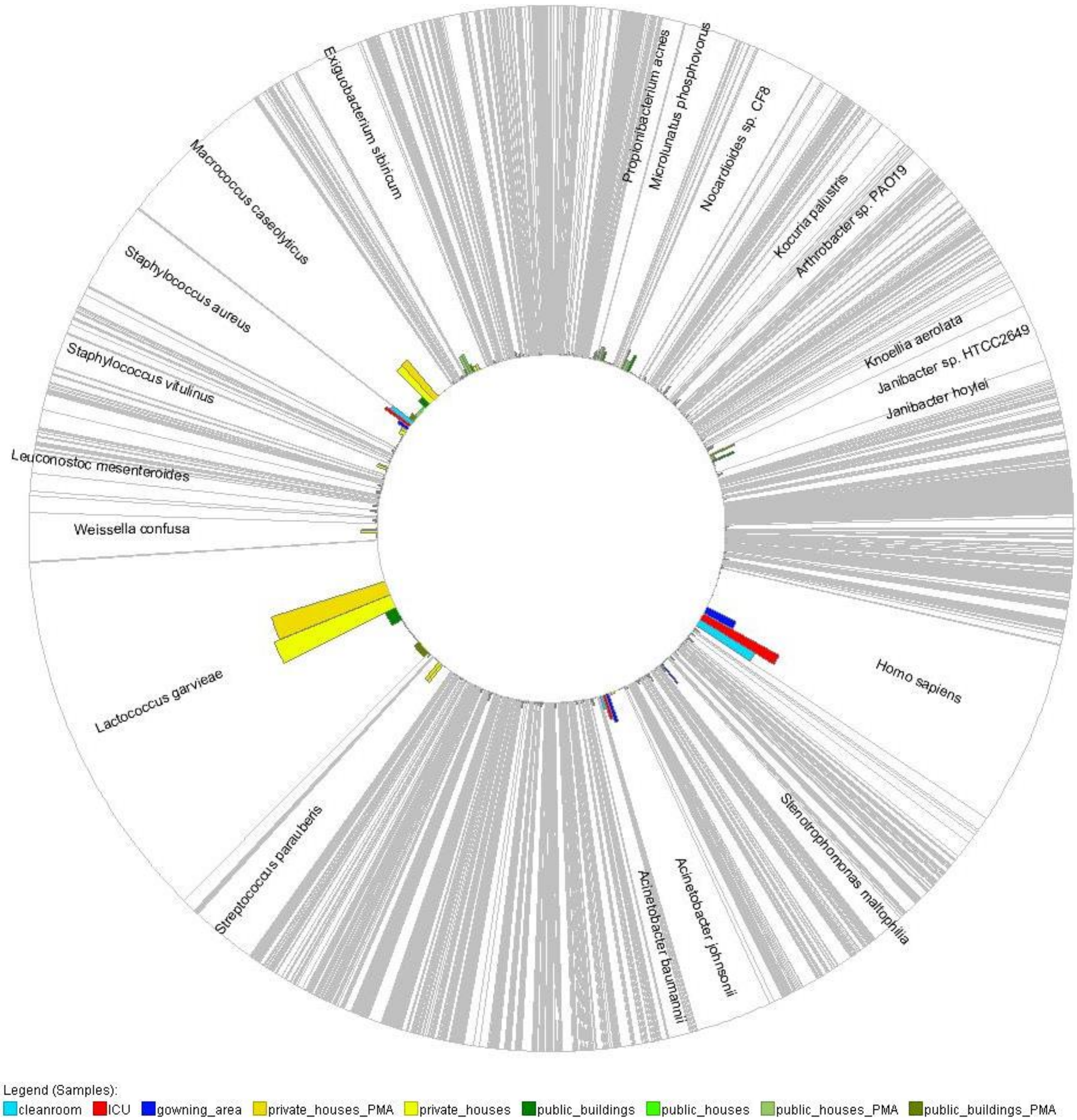

Supplementary Figure 5: **Species profile**

Space filling radial chart of taxa (species level, excluding unassigned reads, normalized, percentage) assigned (BLASTx NCBInr, diamond and rapsearch) to different built environments (MEGAN).

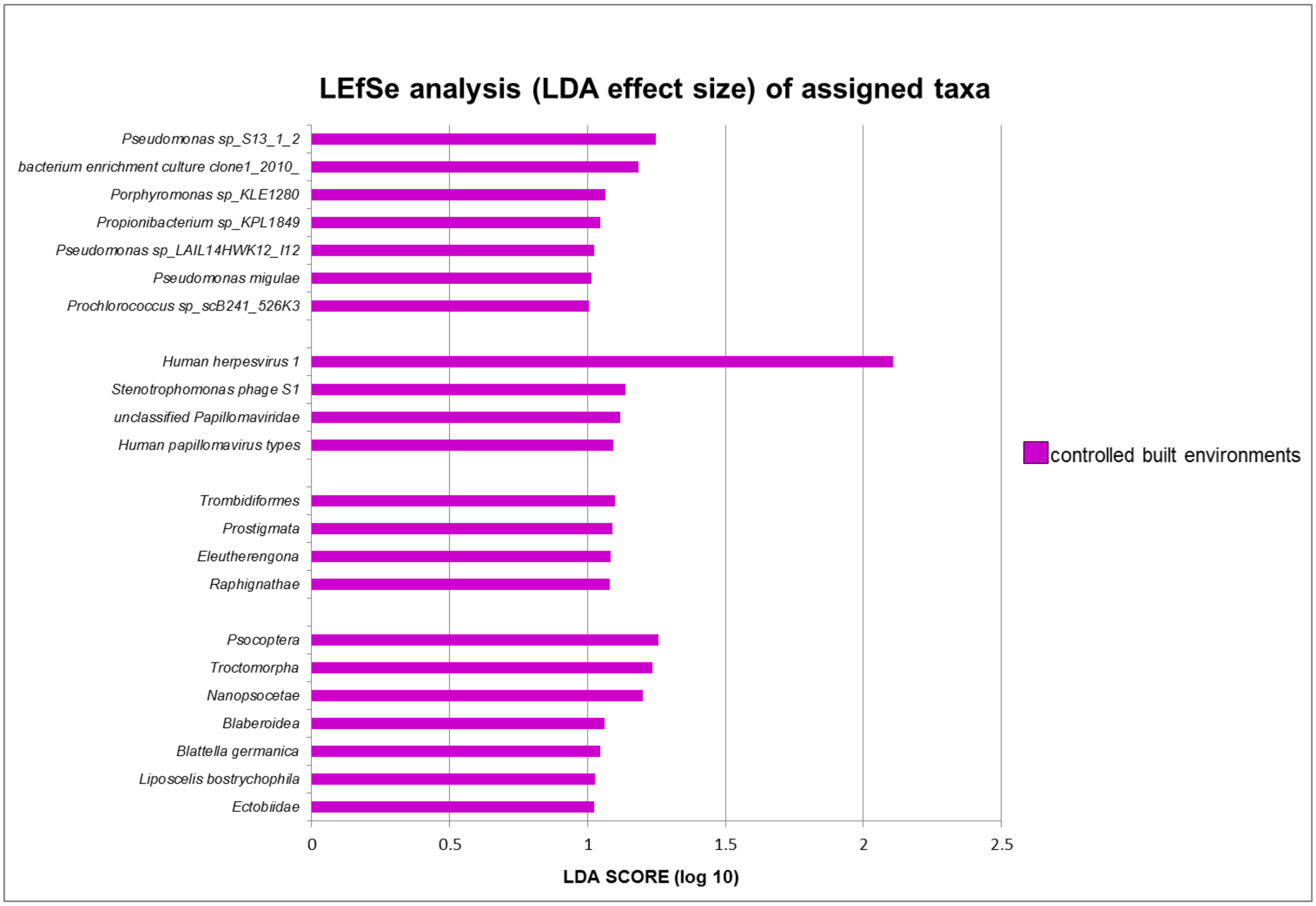

Supplementary Figure 6: **Distinctive taxa of controlled built environments (CB)**

LEfSe analysis (LDA effect size) on taxa (according to NCBInr database) of single reads from metagenomes of CB (ICU, gowning area, cleanroom) and UB (public buildings, public and private houses) built environments with the following parameters: per-sample normalization to 1M, factorial Kruskal-Wallis test among classes (alpha = 0.01), pairwise Wilcoxon test between subclasses (alpha = 0.01), threshold for the LDA score (1.0), strategy for multi-class analysis (all-against-all, more strict).

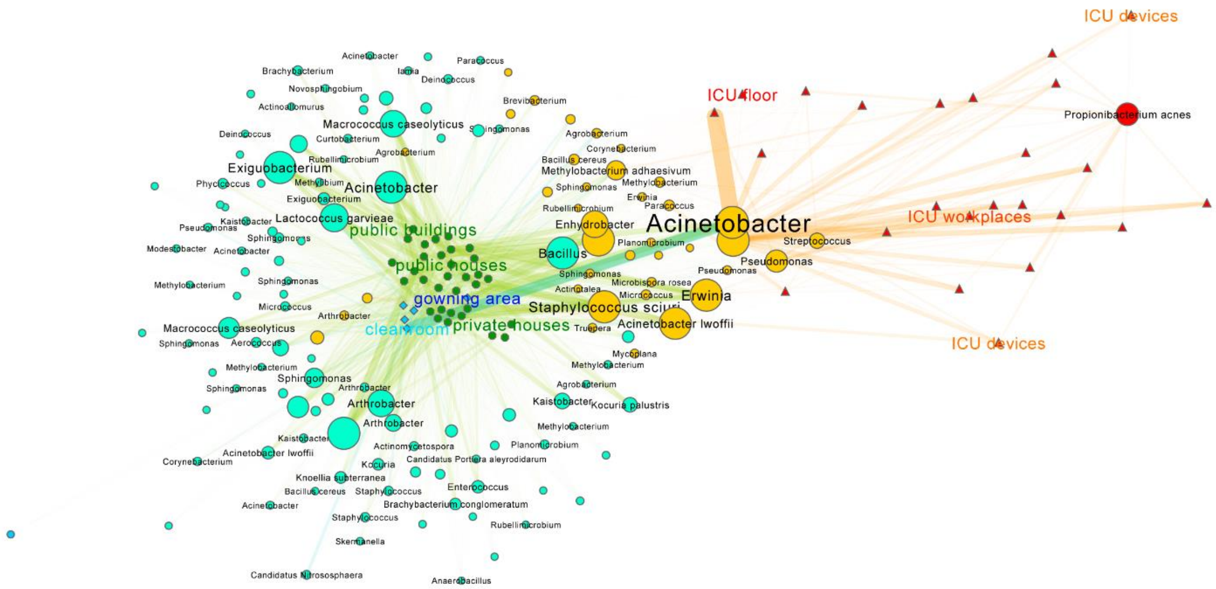

Supplementary Figure 7: **Core microbiome**

Core OTU network based on G-tests for independence of 16S rRNA gene amplicons resolved to genus level. Edge-weighted spring embedded algorithms implemented in Cytoscape were used for visualizations. OTU abundance is reflected by node size. Edge weights by line widths and opacities. Colors refer to different sampled built environments: Cleanroom facility (blue), intensive care unit (red), public buildings, public houses, private houses (all in green).

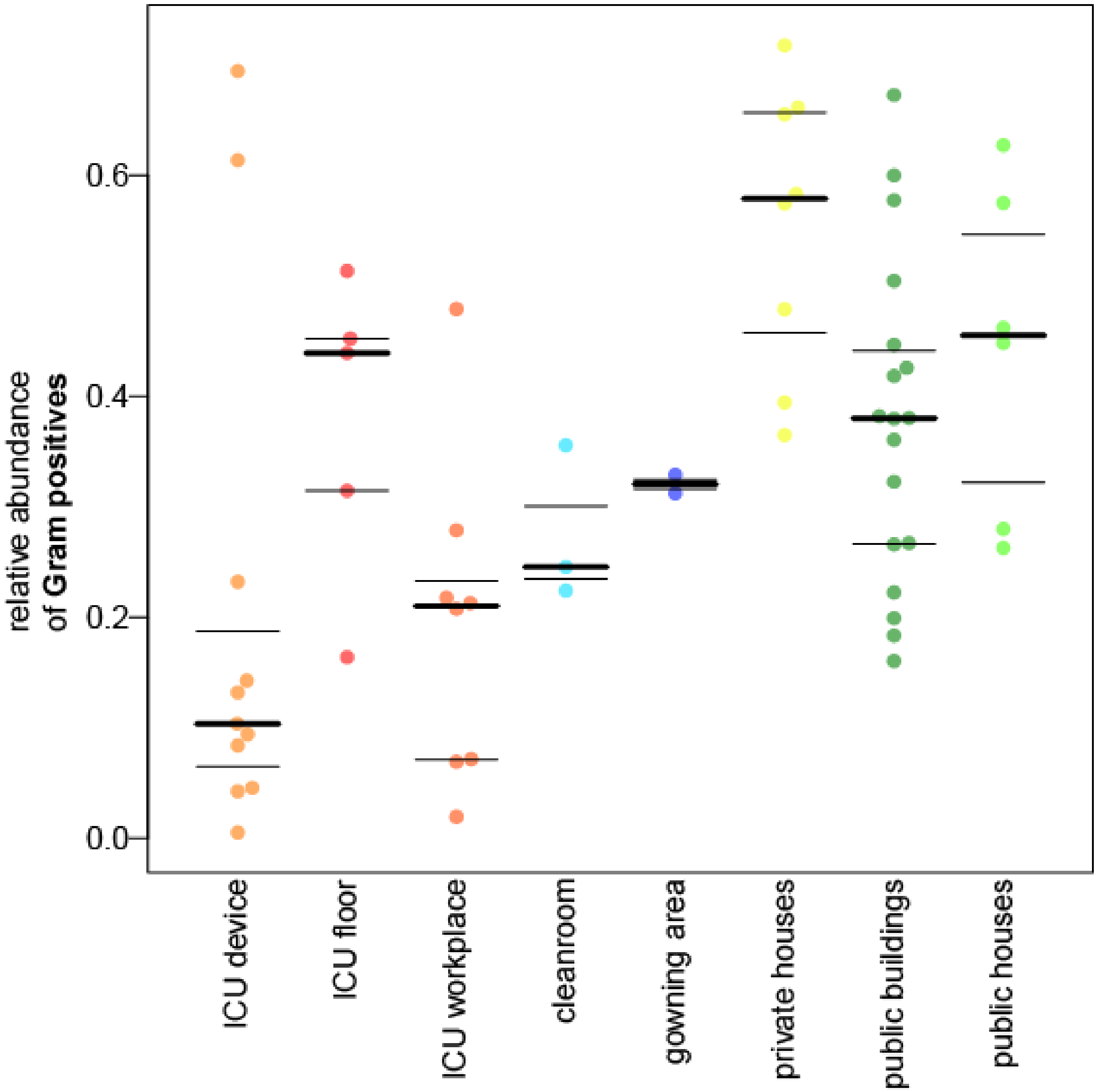

Supplementary Figure 8: **Gram positive bacteria**

Phenotype prediction of Gram positive bacteria based on 16S rRNA gene amplicon analysis.

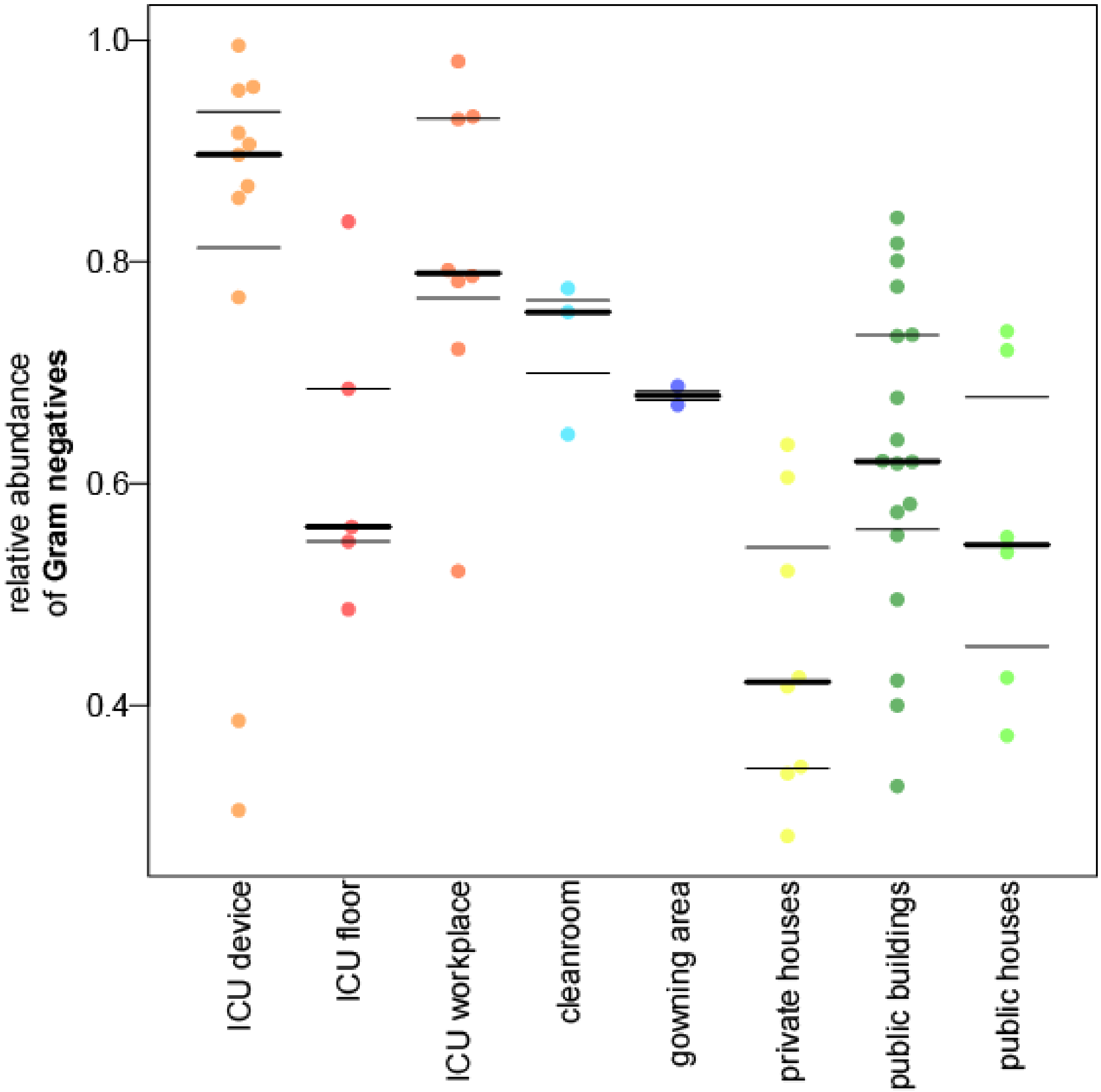

Supplementary Figure 9: **Gram negative bacteria**

Phenotype prediction of Gram negative bacteria based on 16S rRNA gene amplicon analysis.

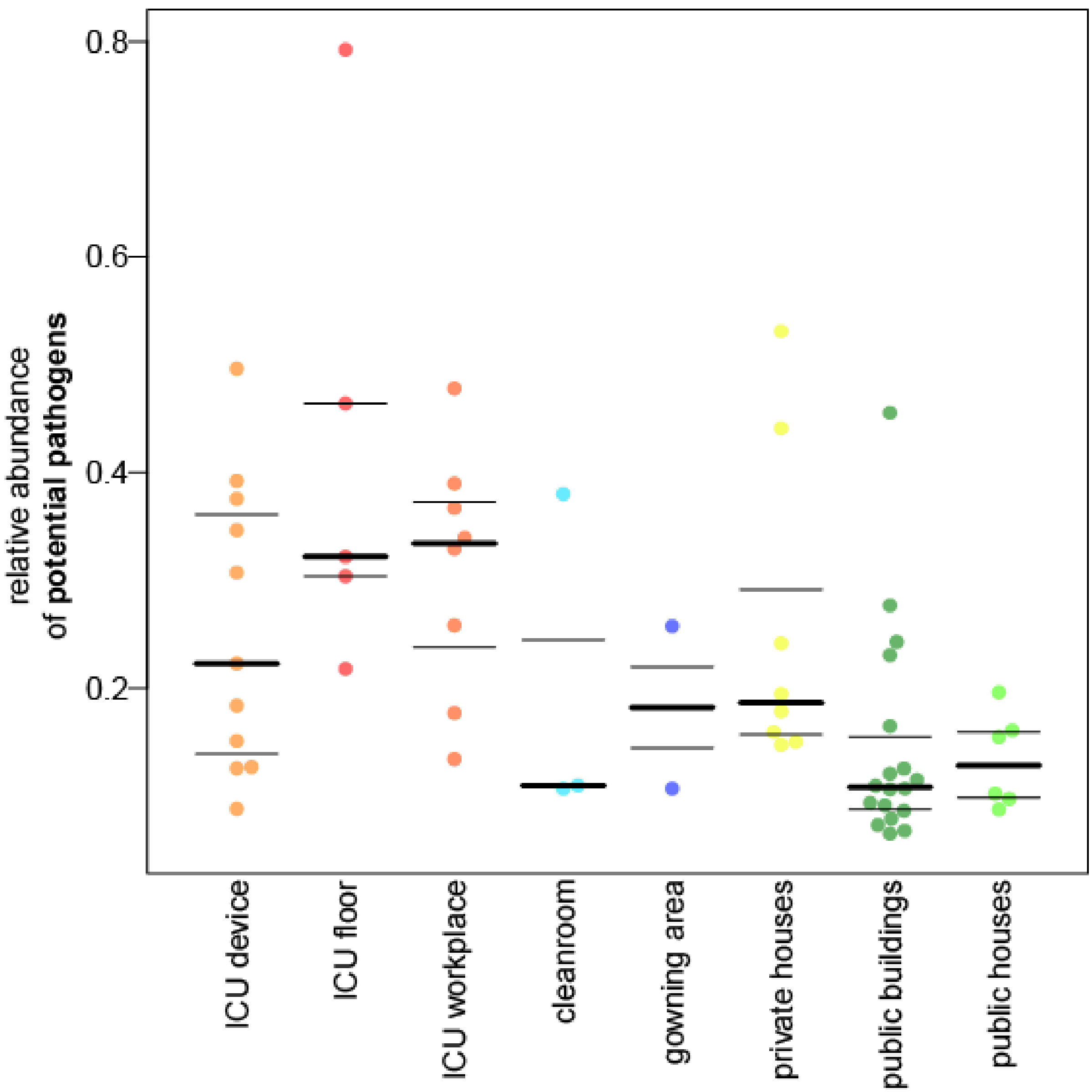

Supplementary Figure 10: **Potential pathogens**

Phenotype prediction of potential pathogens based on 16S rRNA gene amplicon analysis.

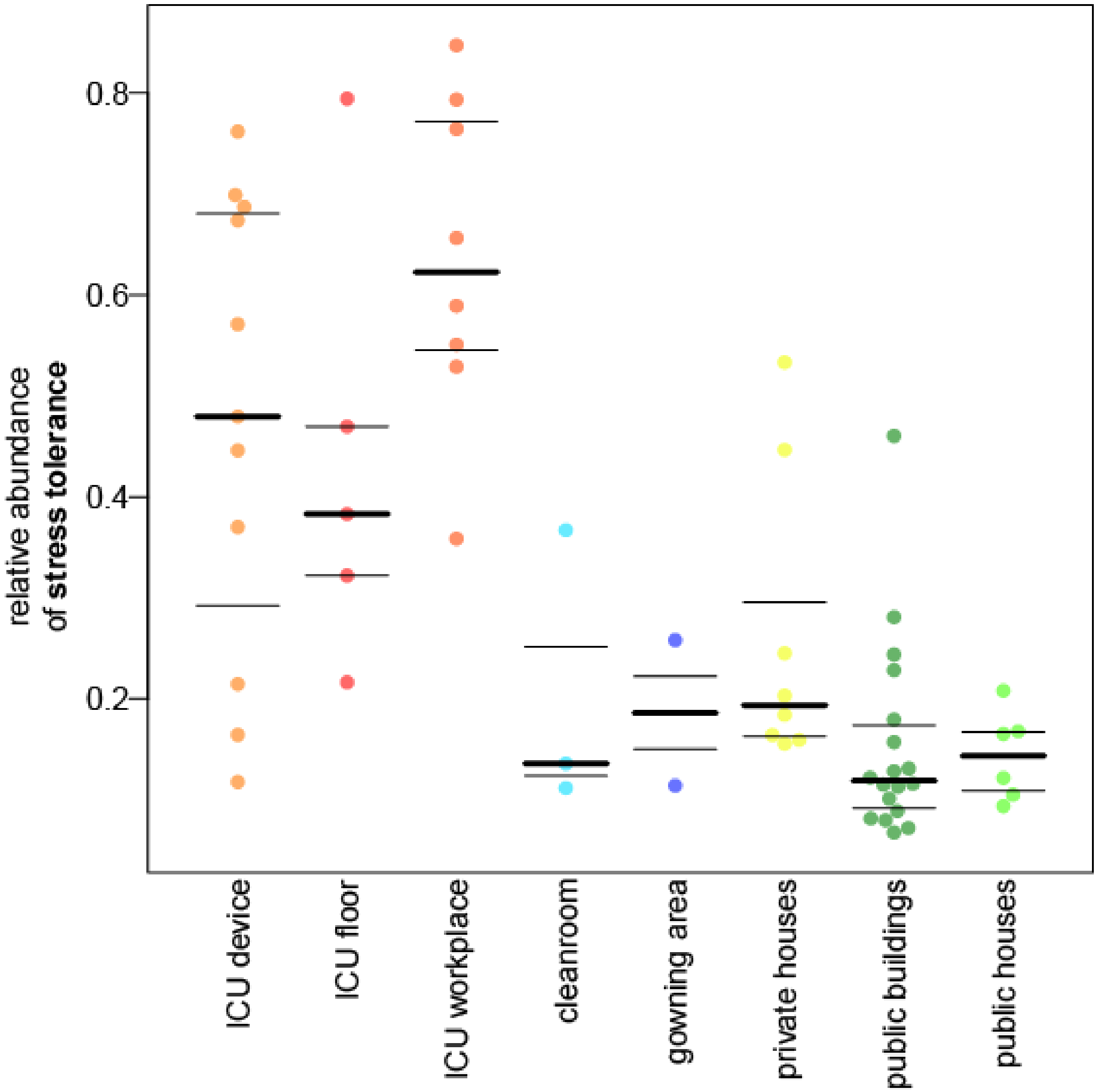

Supplementary Figure 11: **Potential stress tolerance**

Phenotype prediction of potential stress tolerant bacteria based on 16S rRNA gene amplicon analysis.

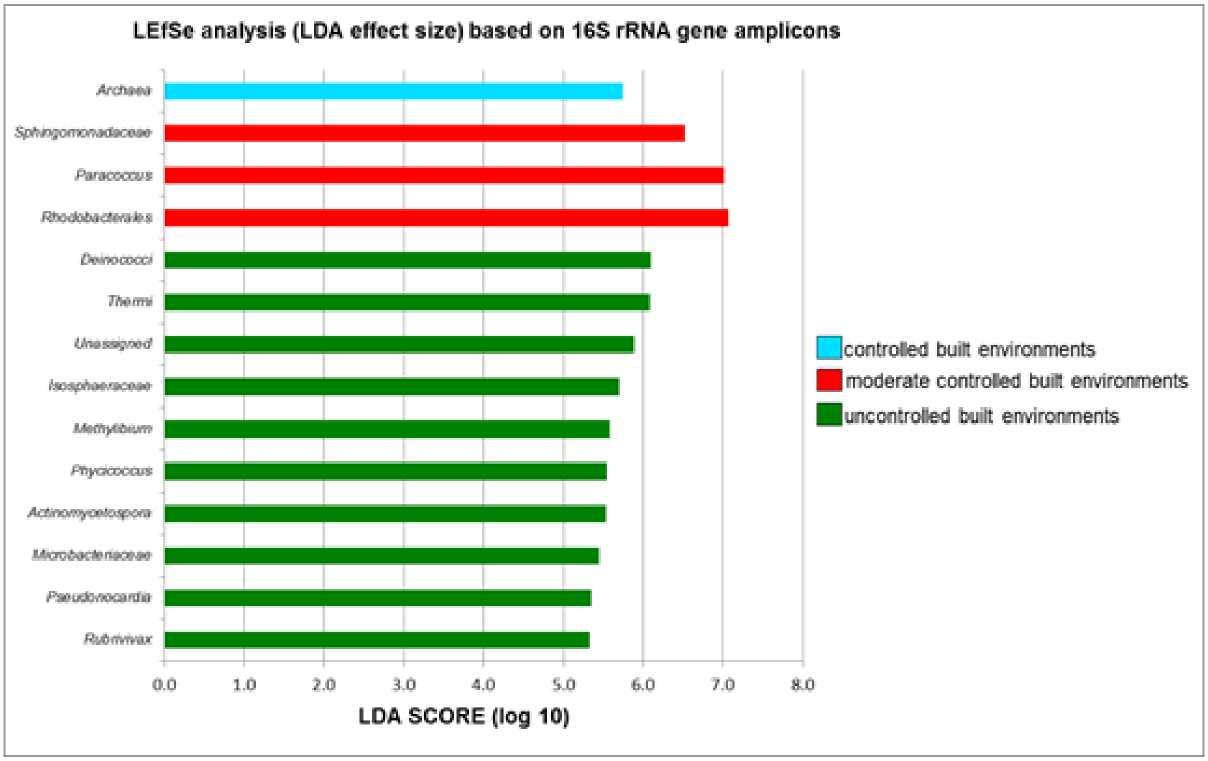

Supplementary Figure 12: **Distinctive taxa based on 16S rRNA gene amplicons**

LEfSe analysis (LDA effect size) on 16S rRNA gene amplicons of controlled (gowning area, cleanroom), moderate controlled (ICU) and uncontrolled (public buildings, public and private houses) built environments with the following parameters: per-sample normalization to 1M, factorial Kruskal-Wallis test among classes (alpha = 0.05), pairwise Wilcoxon test between subclasses (alpha = 0.05), threshold for the LDA score (2.0), strategy for multi-class analysis (all-against-all, more strict).

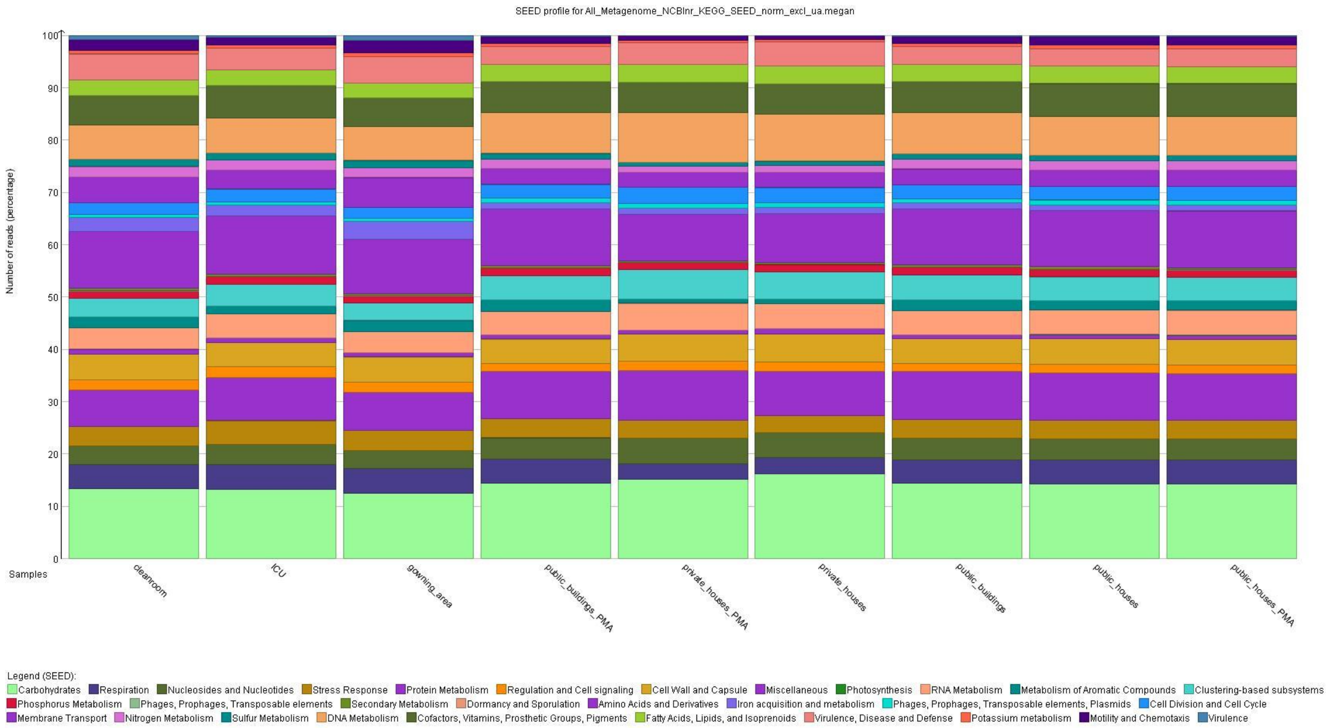

Supplementary Figure 13: **Functional profile (barchart)**

Single reads BLASTx (rapsearch and diamond) vs. NCBI nr. SEED level 1 (derived from MEGAN, excluding unassigned reads, normalized data set).

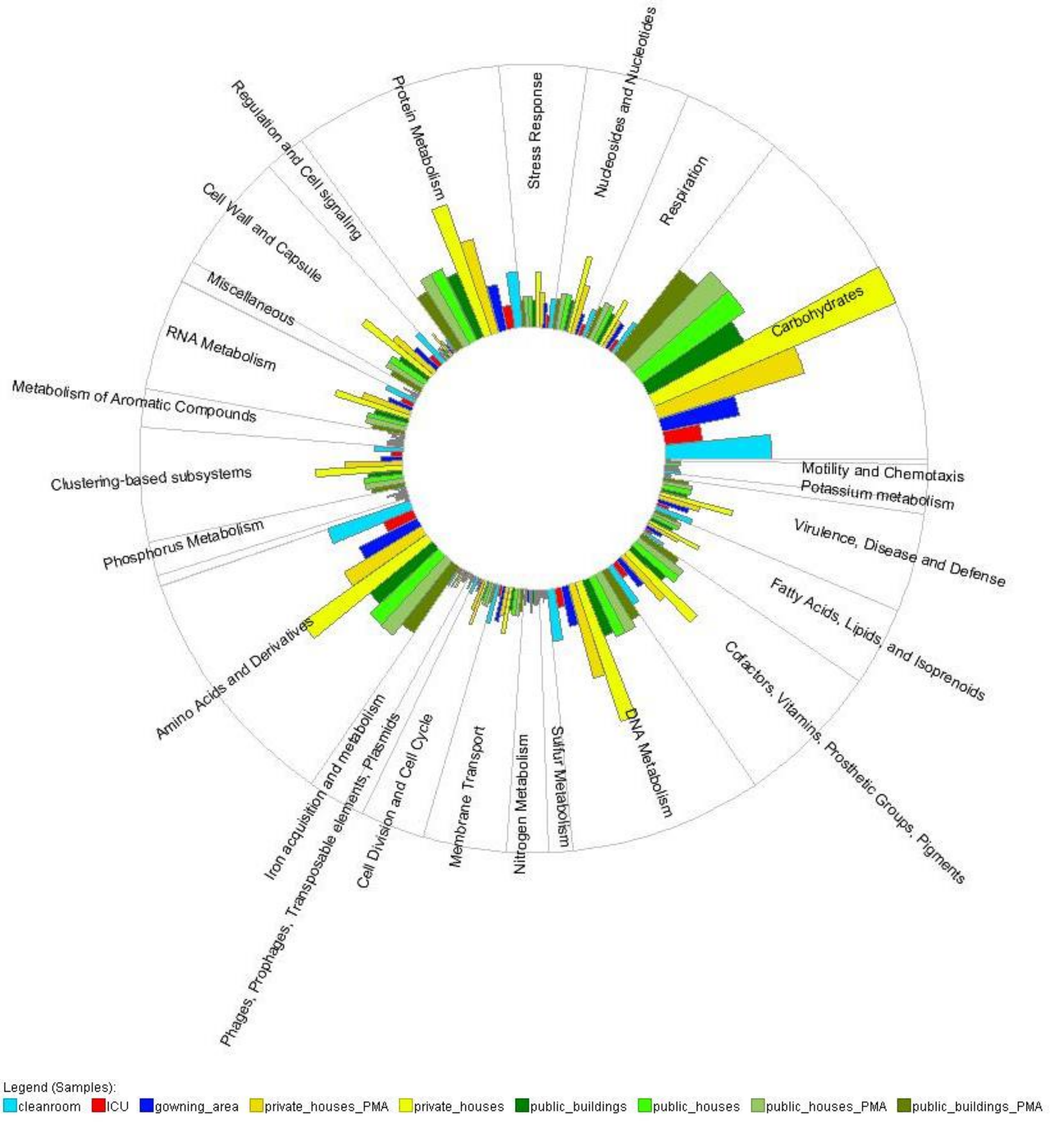

Supplementary Figure 14: **Functional profile (radial chart)**

Space filling radial chart of SEED annotations on level 1 (species level, excluding unassigned reads, normalized, percentage) assigned (BLASTx NCBInr, diamond and rapsearch) to different built environments (MEGAN).

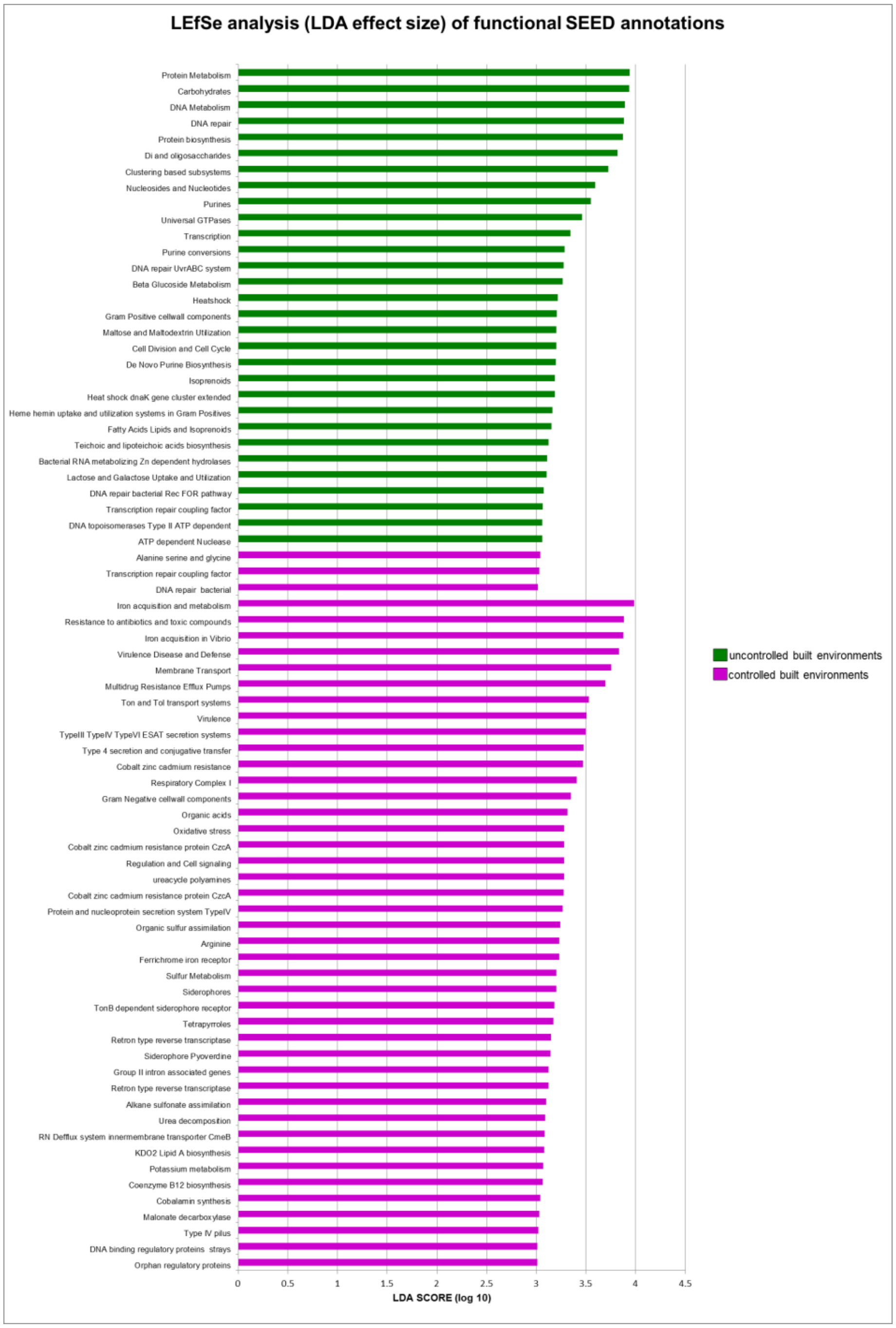

Supplementary Figure 15: **Distinctive functions**

LEfSe analysis (LDA effect size) on functions (according to SEED database) of single reads from metagenomes of CB (ICU, gowning area, cleanroom) and UB (public buildings, public and private houses) built environments with the following parameters: per-sample normalization to 1M, factorial Kruskal-Wallis test among classes (alpha = 0.05), pairwise Wilcoxon test between subclasses (alpha = 0.05), threshold for the LDA score (3.0), strategy for multi-class analysis (all-against-all, more strict).

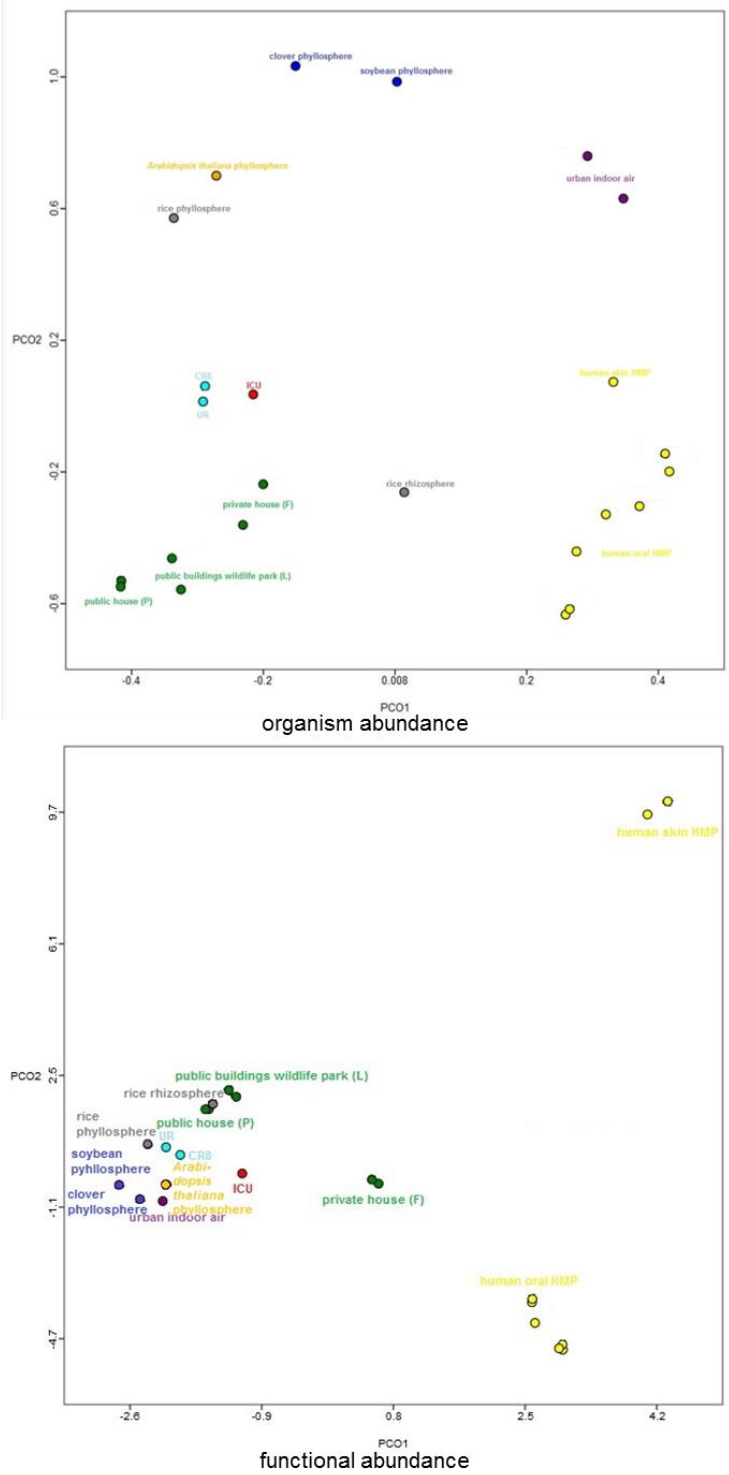

Supplementary Figure 16: **Meta-analysis of organisms and functions**

Comparative analysis of metagenome samples from CB and UB environments with publically available metagenome samples from plants, urban indoor air and the human microbiome project on organism and functional abundance levels visualized through MG-RAST.

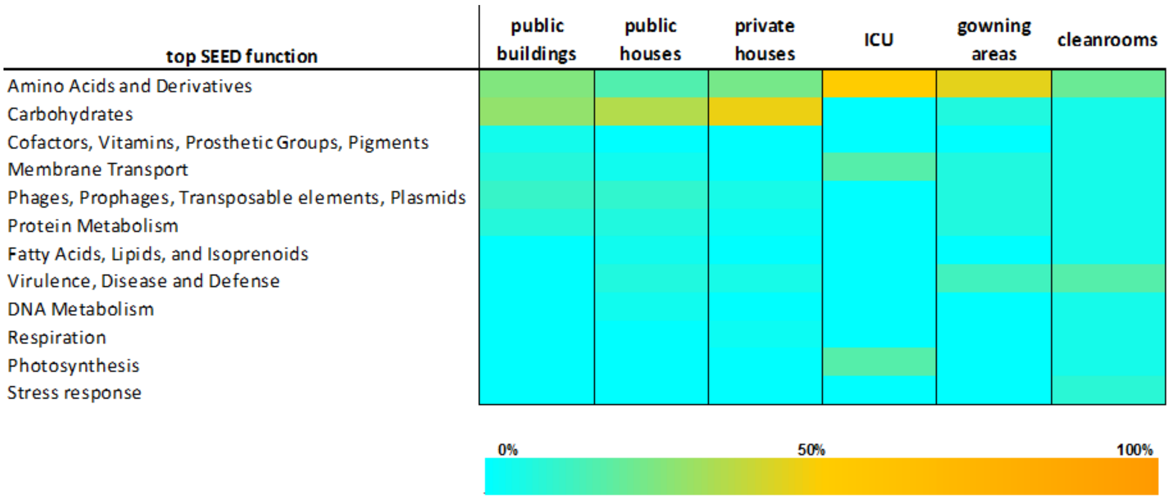

Supplementary Figure 17: **Main genome functions**

Relative proportions of annotated SEED functions with RAST for high quality bins from the metagenomics dataset of all sampled built environments.

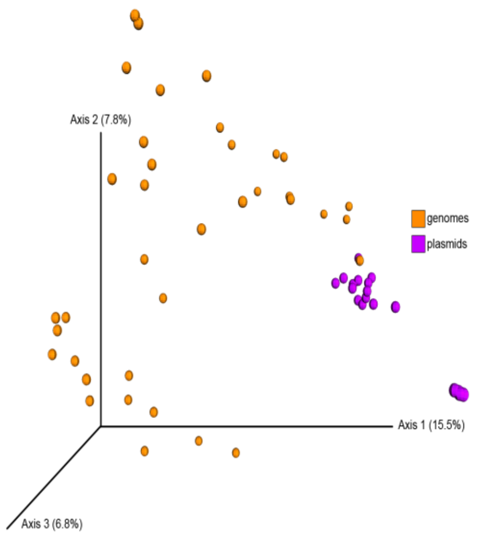

Supplementary Figure 18: **Resistome of genomes and plasmids**

Distances of binned genomes and plasmids according to detected resistance genes (CARD database).

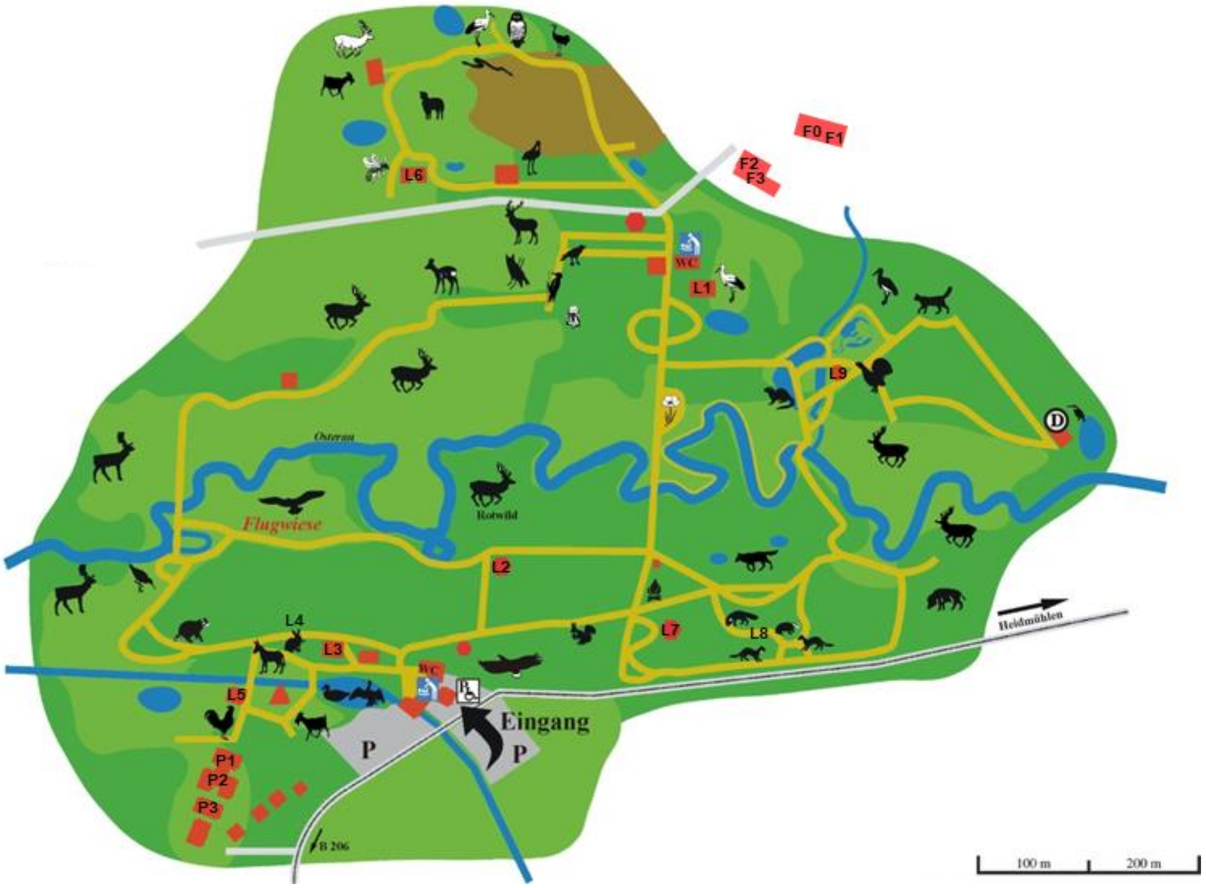

Supplementary Figure 19: **Unrestricted buildings (UB)**

Sampling map of public buildings (L) and public (P) and private houses (F) in a wildlife park in Grossenaspe, Germany (Figure was adapted from https://www.wildpark-eekholt.de/besucherinformationen_lageplan.htm).

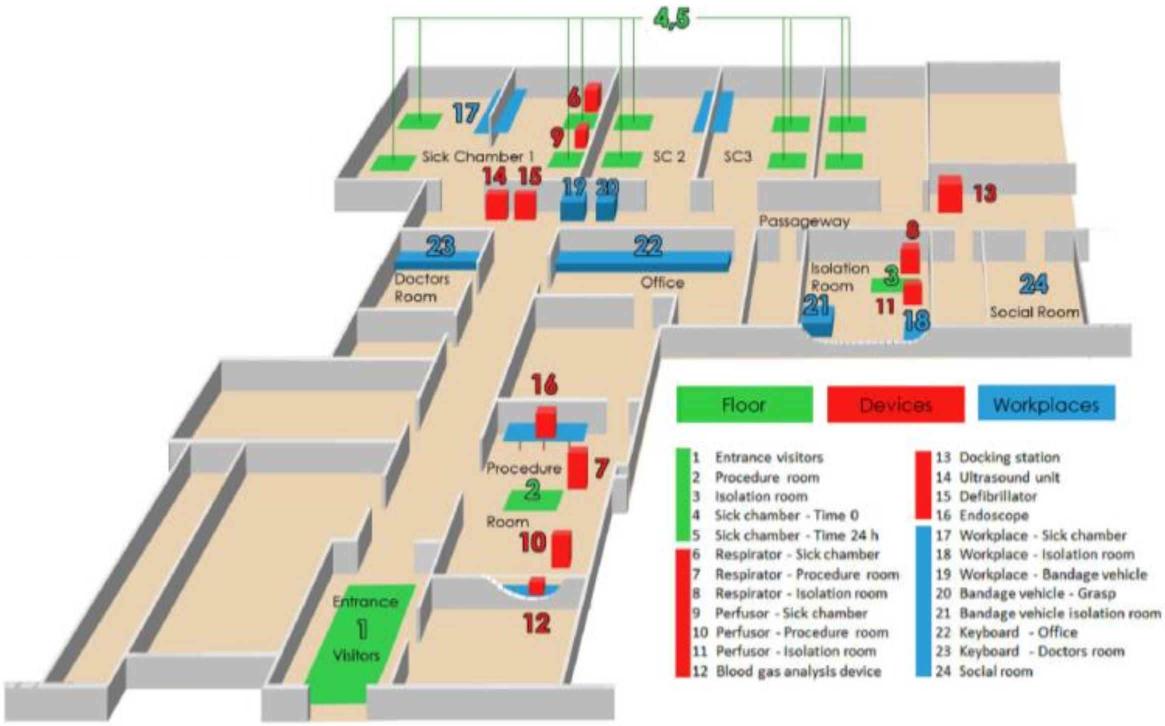

Supplementary Figure 20: **Controlled built environment (CB) - Intensive Care Unit**

Sampling map of the intensive care unit (ICU) at the state hospital in Graz, Austria (Figure was adapted from [1]).

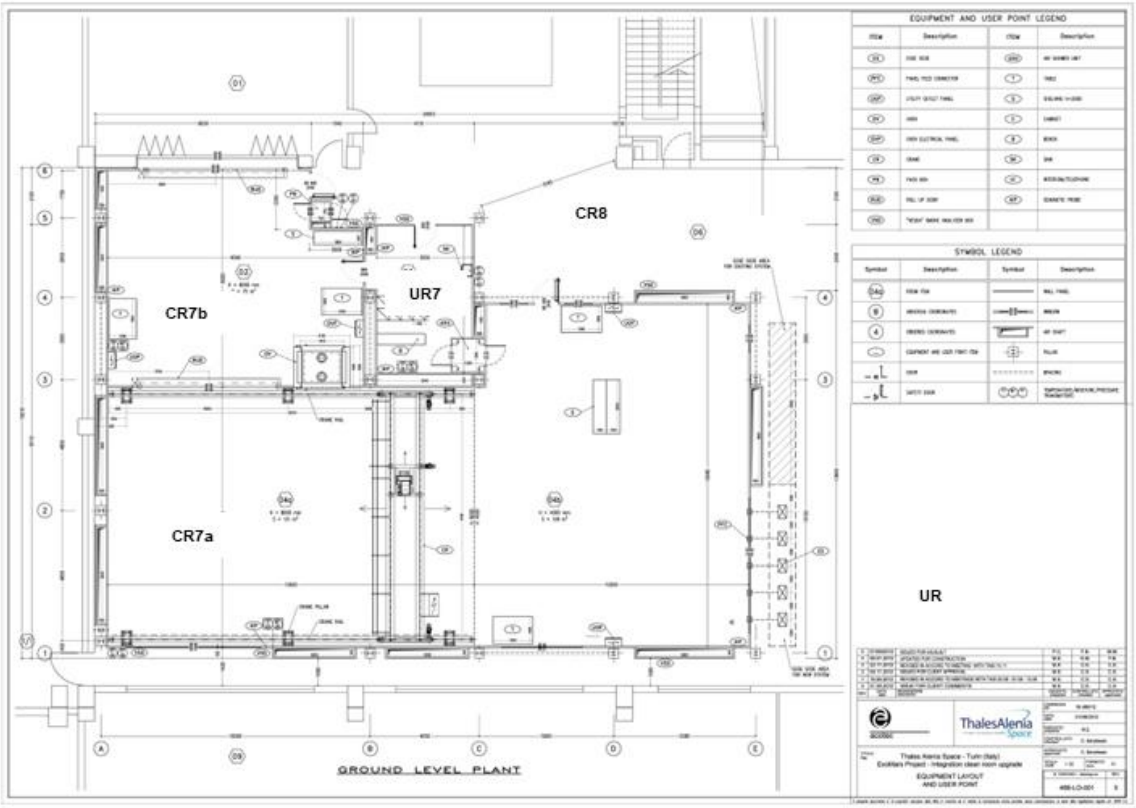

Supplementary Figure 21: **Controlled built environment (CB) - Cleanroom facility**

Sampling map of the Thales Alenia space cleanroom facility in Turin, Italy (Figure was adapted from [2]).

Supplementary Tables

Supplementary Table 1: Alpha diversity estimates from single shotgun reads of the metagenomics dataset against the NCBI nr database using the blastX algorithm.

	Taxa		Taxa (without *Hominoidae*)		KEGG		SEED	
samples	Shannon	Simpson	Shannon	Simpson	Shannon	Simpson	Shannon	Simpson
public_buildings	8.44	68.69	8.44	68.67	11.41	1652.82	10.92	1279.98
public_buildings_PMA	8.42	61.75	8.42	61.73	11.38	1616.20	11.01	1276.63
public_houses	8.97	99.16	8.97	99.08	11.58	1814.65	11.00	1255.54
public_houses_PMA	9.00	101.20	9.00	101.12	11.58	1811.69	11.01	1279.79
private_houses	4.98	6.30	4.98	6.30	11.35	1675.57	10.81	1191.86
private_houses_PMA	5.10	6.78	5.10	6.78	11.27	1591.29	10.97	1223.26
gowning_area	7.76	35.81	8.06	42.62	11.86	1947.20	11.02	884.36
ICU	6.27	12.32	7.28	28.89	12.09	1017.25	11.12	1404.91
cleanroom	7.54	21.66	8.42	56.57	11.90	941.92	11.12	1114.67

Supplementary Table 2: Summary on binned genomes from the shotgun metagenomics data set.

Sample	bin No.	Marker lineage CheckM	# genomes	# markers	# marker sets	0	1	2	3	4	5+	Completeness	Contamination	Strain heterogeneity	binID	Count_Function	Fraction_Function	RAST Function	Count_OTU	Fraction_OTU	RAST OTU	RAST subsystem information
ISO 8 cleanroom (CR8)	10	k__Bacteria (UID203)	5449	102	57	94	8	0	0	0	0	7.5	0	0	CR8_bin10	185	0.069917	Mobile element protein	160	0.171858	Pseudomonas	virulence, disease, and defense
	13	k__Bacteria (UID203)	5449	104	58	72	31	1	0	0	0	33.32	1.72	100	CR8_bin13	11	0.006034	Mobile element protein	184	0.516854	Acinetobacter sp. ATCC	Amino Acids and Derivatives
	16	k__Bacteria (UID1453)	901	171	117	147	21	2	1	0	0	4.17	0.31	20	CR8_bin16	5	0.049505	LSU ribosomal protein L2p (L8e),	1	0.0625	Cyanothece sp PCC 8801	photosynthesis
	21	f__Moraxellaceae (UID4680)	86	689	365	30	560	95	4	0	0	95.05	15.27	94.39	CR8_bin21	141	0.031105	Mobile element protein	43	0.078611	Acinetobacter sp. ATCC	Amino Acids and Derivatives
	27	k__Bacteria (UID203)	5449	103	57	102	1	0	0	0	0	0.16	0	0	CR8_bin27	39	0.04387	Cysteine desulfurase (EC 2.8.1.7), IscS	8	0.020305	Vibrio vulnificus CMCP6	
	3	o__Sphingomonadales (UID3310)	26	569	293	376	178	15	0	0	0	29.25	0.82	40	CR8_bin3	57	0.021261	Mobile element protein	131	0.163138	Sphingobium sp. AP49	virulence, disease, and defense
	31	k__Bacteria (UID203)	5449	103	57	91	12	0	0	0	0	12.2	0	0	CR8_bin31	18	0.00681	Transcriptional regulator, LysR family	370	0.506849	Pseudomonas	Amino Acids and Derivatives
	33	f__Xanthomonadaceae (UID4214)	55	659	290	298	278	76	6	1	0	56.9	16.61	45	CR8_bin33	38	0.005798	Transcriptional regulator, LysR family	1651	0.58298	Stenotrophomonas	Amino Acids and Derivatives
	36	k__Bacteria (UID203)	5449	104	58	102	2	0	0	0	0	0.31	0	0	CR8_bin36	2	0.026667	RND multidrug efflux transporter;	6	0.285714	Micavibrio	Stress response
	39	k__Bacteria (UID203)	5449	103	58	101	2	0	0	0	0	0.34	0	0	CR8_bin39	6	0.034483	Phage tail fiber protein	4	0.070175	Escherichia coli K12	Phage, RNA metabolism
	6	k__Bacteria (UID203)	5449	104	58	94	10	0	0	0	0	12.36	0	0	CR8_bin6	147	0.071118	Mobile element protein	35	0.095368	Acinetobacter	virulence, disease, and defense
	8	o__Actinomycetales (UID1530)	622	259	152	20	216	23	0	0	0	91.67	7.89	100	CR8_bin8	46	0.013154	conserved hypothetical protein	1277	0.740719	Propionibacterium	Carbonhydrates
private houses (F)	1	k__Bacteria (UID203)	5449	104	58	102	2	0	0	0	0	2.59	0	0	F_bin1	16	0.04507	Mobile element protein	28	0.269231	Kocuria rhizophila	Virulence, Disease and Defense (18)
	10	f__Enterobacteriaceae (UID5054)	223	875	305	313	516	46	0	0	0	60.93	5.27	91.3	F_bin10	11	0.003705	diguanylate	305	0.505804	Pantoea ananatis LMG	Amino Acids and Derivatives (140)
	13	o__Lactobacillales (UID544)	293	475	267	268	189	17	1	0	0	46.32	4.01	45	F_bin13	18	0.011159	Mobile element protein	862	0.81015	Enterococcus	Carbohydrates (120)
	15	k__Bacteria (UID203)	5449	104	58	15	24	46	15	3	1	97.65	114.5	21.85	F_bin15	18	0.003797	Oligopeptide ABC transporter,	1639	0.650139	Leuconostoc	Carbohydrates (442)
	16	f__Micrococcaceae (UID1623)	39	457	218	288	145	23	1	0	0	37.4	5.51	15.38	F_bin16	29	0.013507	Mobile element protein	149	0.20781	Micrococcus luteus NCTC	Amino Acids and Derivatives (108)
	17	k__Bacteria (UID203)	5449	104	58	56	30	14	4	0	0	25.31	7.84	61.54	F_bin17	7	0.015184	Mobile element protein	81	0.558621	Enhydrobacter	Respiration (24)
	2	k__Bacteria (UID203)	5449	104	58	43	30	20	7	4	0	84.28	68.1	56.92	F_bin2	89	0.012583	Mobile element protein	139	0.016931		Amino Acids and Derivatives (590)
	20	k__Bacteria (UID203)	5449	104	58	85	19	0	0	0	0	24.64	0	0	F_bin20	8	0.007561	Predicted transcriptional regulators	556	0.827381	Lactococcus garvieae	Carbohydrates (46)
	21	o__Lactobacillales (UID544)	293	475	267	125	221	116	12	1	0	71.87	30.56	13.92	F_bin21	13	0.005066	Lipid A export ATP-binding/permease	818	0.541005	Enterococcus faecalis	Carbohydrates (196)
	23	k__Bacteria (UID203)	5449	104	58	13	24	22	17	15	13	94.98	229.5	30.54	F_bin23	28	0.002748	membrane protein, putative	1180	0.341336	Macrococcus	Carbohydrates (1283)
	25	k__Bacteria (UID203)	5449	104	58	97	7	0	0	0	0	8.62	0	0	F_bin25	2	0.006667	Proposed peptidoglycan lipid II	118	0.819444	Enhydrobacter	Amino Acids and Derivatives (12)
	29	k__Bacteria (UID203)	5449	104	58	96	8	0	0	0	0	1.96	0	0	F_bin29	2	0.037037	Pyruvate dehydrogenase E1	2	0.285714	Exiguobacterium	
	31	c__Bacilli (UID259)	750	273	152	65	71	82	35	19	1	84.18	93.53	84.89	F_bin31	42	0.005469	acetyltransferase, GNAT family	4665	0.847411	Exiguobacterium	Amino Acids and Derivatives (631)
	33	k__Bacteria (UID203)	5449	104	58	44	40	17	3	0	0	49.06	14.13	38.46	F_bin33	12	0.007417	Oligopeptide ABC transporter,	91	0.232737	Weissella confusa LBAE	Carbohydrates (104)
	34	c__Bacilli (UID285)	586	325	181	42	225	57	1	0	0	93.38	20.3	90	F_bin34	6	0.00274	Lipid A export ATP-binding/permease	97	0.211329	Macrococcus	Carbohydrates (176)
	35	k__Bacteria (UID203)	5449	104	58	0	5	76	13	10	0	100	130.27	32	F_bin35	87	0.014897	Mobile element protein	1242	0.513223	Lactococcus garvieae	Carbohydrates (753)
	36	k__Bacteria (UID203)	5449	103	57	69	12	5	10	7	0	11.93	7.72	24.68	F_bin36	4	0.04	LSU ribosomal protein L18p (L5e)	2	0.222222	Bacillus coahuilensis m4-	
	4	k__Bacteria (UID203)	5449	104	58	94	8	2	0	0	0	7.52	1.72	0	F_bin4	13	0.02305	Mobile element protein	17	0.084577	Leuconostoc	Phages, Prophages, Transposable
	7	o__Actinomycetales (UID1530)	622	257	151	155	99	3	0	0	0	36.89	1.99	100	F_bin7	5	0.004386	Acetyl-coenzyme A synthetase (EC	104	0.314199	Rothia mucilaginosa	Carbohydrates (47)
	8	k__Bacteria (UID203)	5449	104	58	24	33	23	14	8	2	83.54	86.21	30.08	F_bin8	139	0.016931	Mobile element protein	1853	0.538663	Staphylococcus aureus	Carbohydrates (580)
private houses PMA (F_plus)	0	k__Bacteria (UID203)	5449	103	57	97	6	0	0	0	0	4.7	0	0	F_plus_bin0	2	0.019608	Mobile element protein	47	0.810345	Exiguobacterium	
	10	o__Lactobacillales (UID374)	471	348	189	55	225	65	3	0	0	95.08	22.4	78.38	F_plus_bin10	33	0.013121	Mobile element protein	1549	0.838657	Leuconostoc	Amino Acids and Derivatives (258)
	12	k__Bacteria (UID203)	5449	104	58	62	9	11	13	1	8	15.44	21.65	14.76	F_plus_bin12	8	0.024169	SSU ribosomal protein S17p (S11e)	6	0.111111	Lactococcus garvieae	Protein Metabolism (7)
	13	k__Bacteria (UID203)	5449	103	57	4	46	44	7	2	0	95.45	44.58	57.14	F_plus_bin13	19	0.005522	6-phospho-beta-glucosidase (EC	449	0.487514	Weissella confusa LBAE	Carbohydrates (297)
	14	o__Lactobacillales (UID543)	294	472	265	34	347	81	6	4	0	98.24	26.26	91.06	F_plus_bin14	28	0.010068	Mobile element protein	493	0.644444	Streptococcus iniae SF1	Carbohydrates (414)
	16	f__Enterobacteriaceae (UID5054)	223	875	305	341	494	40	0	0	0	54.71	3.42	92.5	F_plus_bin16	14	0.004848	diguanylate	248	0.438163	Pantoea ananatis LMG	Amino Acids and Derivatives (111)
	18	k__Bacteria (UID203)	5449	103	57	55	21	22	5	0	0	71.93	49.12	94.59	F_plus_bin18	39	0.005431	acetyltransferase, GNAT family	4405	0.843385	Exiguobacterium	Amino Acids and Derivatives (606)
	19	k__Bacteria (UID203)	5449	104	58	65	34	5	0	0	0	51.1	8.62	100	F_plus_bin19	8	0.004499	Cell division protein FtsK	816	0.828426	Macrococcus	Carbohydrates (68)
	20	c__Gammaproteobacteria (UID4201)	1164	275	174	203	70	2	0	0	0	23.53	1.15	100	F_plus_bin20	11	0.016667	Mobile element protein	196	0.710145	Enhydrobacter	Virulence, Disease and Defense (30)
	21	k__Bacteria (UID203)	5449	104	58	61	29	11	3	0	0	47.79	18.57	0	F_plus_bin21	40	0.013717	Mobile element protein	516	0.321696	Lactococcus garvieae	Carbohydrates (217)
	23	c__Bacilli (UID285)	586	325	181	51	233	41	0	0	0	92.81	14.55	87.8	F_plus_bin23	6	0.002668	Lipid A export ATP-binding/permease	92	0.192469	Macrococcus	Carbohydrates (195)
	30	c__Gammaproteobacteria (UID4201)	1164	275	174	40	92	95	39	7	2	91.06	84.52	55.11	F_plus_bin30	83	0.013291	Mobile element protein	77	0.106796	Acinetobacter sp. ATCC	Amino Acids and Derivatives (440)
	32	k__Bacteria (UID203)	5449	104	58	69	22	12	1	0	0	37.46	15.83	13.33	F_plus_bin32	17	0.009346	Mobile element protein	126	0.208264	Kocuria palustris PEL	Carbohydrates (61)
	35	k__Bacteria (UID203)	5449	103	57	98	5	0	0	0	0	0.99	0	0	F_plus_bin35	3	0.044776	Mobile element protein	6	0.206897	Exiguobacterium	Phages, Prophages, Transposable
	37	o__Lactobacillales (UID543)	294	469	262	69	371	29	0	0	0	86.45	6.54	96.55	F_plus_bin37	17	0.009403	Mobile element protein	963	0.833766	Lactococcus garvieae	Carbohydrates (156)
	38	k__Bacteria (UID203)	5449	104	58	83	21	0	0	0	0	19.51	0	0	F_plus_bin38	5	0.007657	Lipid A export ATP-binding/permease	363	0.862233	Enterococcus	Carbohydrates (43)
	4	k__Bacteria (UID203)	5449	104	58	21	33	24	19	5	2	84.77	89.99	29.01	F_plus_bin4	124	0.016286	Mobile element protein	1634	0.530864	Staphylococcus aureus	Carbohydrates (610)
	7	o__Actinomycetales (UID1530)	622	257	151	169	86	2	0	0	0	29.67	0.99	100	F_plus_bin7	5	0.005568	Methionine ABC transporter ATP-	78	0.304688	Rothia mucilaginosa	Amino Acids and Derivatives (45)
	9	k__Bacteria (UID203)	5449	104	58	8	31	29	25	10	1	97.18	138.04	39.11	F_plus_bin9	31	0.003972	membrane protein, putative	768	0.345324	Staphylococcus aureus	Carbohydrates (955)
ICU	1	k__Bacteria (UID203)	5449	103	58	102	1	0	0	0	0	0.16	0	0	ICU_bin1	1	0.2	transposase	0	0	0	
	18	g__Pseudomonas (UID4525)	39	1389	369	93	1270	24	2	0	0	93.47	2.51	26.67	ICU_bin18	66	0.008586	Mobile element protein	709	0.387009	Pseudomonas	Amino Acids and Derivatives (639)
	19	root (UID1)	5656	56	24	55	1	0	0	0	0	1.04	0	0	ICU_bin19	3	0.022388	Plasmin, protease S01.233	4	0.057143	Pseudomonas	RNA Metabolism (1)
	20	k__Bacteria (UID203)	5449	103	58	97	6	0	0	0	0	2.59	0	0	ICU_bin20	2	0.071429	DNA-directed RNA polymerase beta	1	0.25	Leuconostoc	
	23	k__Bacteria (UID203)	5449	104	58	100	4	0	0	0	0	2.19	0	0	ICU_bin23	10	0.042017	Mobile element protein	9	0.125	Pseudomonas	Membrane Transport (10)
	24	f__Moraxellaceae (UID4680)	86	689	365	18	662	9	0	0	0	97.59	0.59	0	ICU_bin24	217	0.058808	Mobile element protein	52	0.106776	Acinetobacter	Amino Acids and Derivatives (336)
	3	o__Actinomycetales (UID1530)	622	258	151	17	204	37	0	0	0	92.19	14.29	100	ICU_bin3	46	0.013699	conserved hypothetical protein	1230	0.750916	Propionibacterium	Amino Acids and Derivatives (255)
	7	k__Bacteria (UID1453)	901	171	117	148	21	2	0	0	0	3.74	0.52	0	ICU_bin7	3	0.032609	photosystem I assembly related	1	0.166667	Prevotella sp. oral taxon	Photosynthesis (17)
public buildings (L_clean)	0	f__Micrococcaceae (UID1623)	39	457	218	35	356	64	2	0	0	94.1	14.79	92.86	L_bin0	15	0.003896	Aldehyde dehydrogenase (EC 1.2.1.3)	506	0.397799	Arthrobacter arilaitensis	Amino Acids and Derivatives (389)
	1	c__Bacilli (UID259)	750	273	152	210	62	1	0	0	0	6.85	0.06	100	L_bin1	3	0.017241	Phage terminase, large subunit	48	0.648649	Exiguobacterium	Phages, Prophages, Transposable
	10	c__Bacilli (UID285)	586	325	181	8	300	12	5	0	0	96.13	7.6	92.59	L_bin10	6	0.002725	RND multidrug efflux transporter;	93	0.197872	Macrococcus	Protein Metabolism (208)
	13	k__Bacteria (UID203)	5449	104	58	74	28	2	0	0	0	45.69	3.45	100	L_bin13	12	0.003131	ABC transporter ATP-binding protein	1054	0.594138	Arthrobacter aurescens	Carbohydrates (224)
	14	k__Bacteria (UID203)	5449	103	57	82	17	2	2	0	0	18.42	0.96	12.5	L_bin14	9	0.007098	Mobile element protein	125	0.259875	Brachybacterium	Membrane Transport (21)
	15	k__Bacteria (UID203)	5449	104	58	92	8	4	0	0	0	2.45	0.63	0	L_bin15	5	0.032895	Mobile element protein	8	0.145455	Bacillus anthracis str.	Phages, Prophages, Transposable
	16	f__Micrococcaceae (UID1623)	39	457	218	220	233	4	0	0	0	52.37	1.16	100	L_bin16	18	0.005513	Aldehyde dehydrogenase (EC 1.2.1.3)	97	0.117291	Arthrobacter aurescens	Amino Acids and Derivatives (134)
	17	c__Bacilli (UID259)	750	273	152	75	105	68	22	3	0	85.8	57.95	87.5	L_bin17	30	0.005201	acetyltransferase, GNAT family	3553	0.842942	Exiguobacterium	Amino Acids and Derivatives (514)
	18	f__Micrococcaceae (UID1631)	31	367	171	263	90	13	1	0	0	25.45	3.96	50	L_bin18	17	0.006474	Beta-galactosidase (EC 3.2.1.23)	741	0.607875	Arthrobacter aurescens	Cofactors, Vitamins, Prosthetic
	4	k__Bacteria (UID203)	5449	104	58	74	19	9	2	0	0	43.1	20.69	93.33	L_bin4	47	0.006359	Mobile element protein	1784	0.547743	Janibacter sp. HTCC2649	Carbohydrates (342)
	6	o__Actinomycetales (UID1593)	69	400	198	320	79	1	0	0	0	18.37	0.51	100	L_bin6	4	0.006126	3-oxoacyl-[acyl-carrier protein]	10	0.064516	Burkholderia	Membrane Transport (20)
	7	k__Bacteria (UID203)	5449	104	58	21	37	20	16	9	1	71.26	43.71	11.36	L_bin7	37	0.011534	Mobile element protein	557	0.359587	Enterococcus	Carbohydrates (146)
public buildings PMA (L_plus)	10	f__Micrococcaceae (UID1623)	39	457	218	177	274	6	0	0	0	64.26	1.62	83.33	L_plus_bin10	18	0.00483	Aldehyde dehydrogenase (EC 1.2.1.3)	108	0.115756	Arthrobacter aurescens	Amino Acids and Derivatives (211)
	11	k__Bacteria (UID203)	5449	104	58	2	80	22	0	0	0	98.12	18.65	100	L_plus_bin11	8	0.003226	Mobile element protein	101	0.191651	Macrococcus	Protein Metabolism (246)
	13	o__Lactobacillales (UID544)	293	475	267	301	151	14	8	1	0	32.93	3.51	18.18	L_plus_bin13	12	0.009119	Mobile element protein	530	0.703851	Enterococcus	Carbohydrates (36)
	14	k__Bacteria (UID203)	5449	104	58	85	16	3	0	0	0	29.31	5.17	100	L_plus_bin14	15	0.003315	Butyryl-CoA dehydrogenase (EC	1029	0.513217	Janibacter sp. HTCC2649	Carbohydrates (223)
	16	c__Bacilli (UID259)	750	273	152	39	131	86	15	2	0	93.41	56.03	92.31	L_plus_bin16	23	0.004423	acetyltransferase, GNAT family	3258	0.852656	Exiguobacterium	Amino Acids and Derivatives (422)
	17	k__Bacteria (UID203)	5449	104	58	59	41	4	0	0	0	63.11	5.17	100	L_plus_bin17	20	0.003972	Beta-glucosidase (EC 3.2.1.21)	1378	0.59835	Arthrobacter aurescens	Carbohydrates (338)
	18	k__Bacteria (UID203)	5449	103	57	71	14	13	5	0	0	12.6	5.98	0	L_plus_bin18	6	0.017442	Replicative DNA helicase (EC 3.6.1.-);	16	0.140351	Bacillus anthracis str.	Phages, Prophages, Transposable
	4	o__Actinomycetales (UID1593)	69	400	198	283	111	6	0	0	0	26.37	1.19	50	L_plus_bin4	6	0.004946	Alpha-glucosidase (EC 3.2.1.20)	22	0.071429	Microbacterium	Carbohydrates (30)
	6	f__Micrococcaceae (UID1623)	39	457	218	54	355	47	1	0	0	89.88	8.8	78	L_plus_bin6	19	0.005278	Aldehyde dehydrogenase (EC 1.2.1.3)	468	0.397621	Arthrobacter arilaitensis	Amino Acids and Derivatives (380)
	7	k__Bacteria (UID203)	5449	104	58	78	23	3	0	0	0	31.11	3.61	33.33	L_plus_bin7	13	0.004779	FIG00761799: membrane protein	544	0.503238	Brachybacterium	Carbohydrates (76)
	8	k__Bacteria (UID203)	5449	104	58	43	26	27	7	1	0	55.09	39.08	24.07	L_plus_bin8	40	0.004997	Mobile element protein	1450	0.456405	Janibacter sp. HTCC2649	Amino Acids and Derivatives (284)
	9	f__Micrococcaceae (UID1631)	31	367	171	277	77	12	1	0	0	25.17	4.48	53.33	L_plus_bin9	41	0.017402	Mobile element protein	462	0.469512	Arthrobacter aurescens	Carbohydrates (62)
public houses (P)	0	root (UID1)	5656	56	24	55	1	0	0	0	0	4.17	0	0	P_bin0	5	0.006935	Beta-glucosidase (EC 3.2.1.21)	13	0.068783	Janibacter sp. HTCC2649	Carbohydrates (10)
	1	k__Bacteria (UID203)	5449	104	58	100	3	0	1	0	0	6.9	3.45	33.33	P_bin1	65	0.05893	Mobile element protein	53	0.161585	Kocuria rhizophila	Virulence, Disease and Defense (33)
	10	o__Actinomycetales (UID1530)	622	257	152	27	212	16	2	0	0	91.89	10.2	100	P_bin10	50	0.01197	Mobile element protein	457	0.322512	Kocuria rhizophila	Amino Acids and Derivatives (321)
	13	k__Bacteria (UID203)	5449	104	58	53	36	13	1	1	0	44.37	10.19	68.18	P_bin13	18	0.00542	Methyl-accepting chemotaxis protein I	247	0.346424	Pantoea ananatis LMG	Carbohydrates (113)
	14	c__Bacilli (UID259)	750	273	152	13	124	108	28	0	0	96.05	70.95	94.27	P_bin14	38	0.00598	acetyltransferase, GNAT family	3900	0.844704	Exiguobacterium	Amino Acids and Derivatives (541)
	15	f__Enterobacteriaceae (UID5054)	223	874	303	122	420	284	47	1	0	87.38	44.72	50.81	P_bin15	22	0.003578	Methyl-accepting chemotaxis protein I	213	0.184896	Pantoea ananatis LMG	Carbohydrates (430)
	16	f__Micrococcaceae (UID1623)	39	457	218	166	283	8	0	0	0	64.59	1.47	62.5	P_bin16	10	0.004045	Aldehyde dehydrogenase (EC 1.2.1.3)	355	0.433455	Arthrobacter arilaitensis	Carbohydrates (180)
	18	o__Actinomycetales (UID1530)	622	257	152	19	207	26	5	0	0	93.33	18.55	95.12	P_bin18	19	0.004781	Mobile element protein	505	0.385791	Kocuria palustris PEL	Amino Acids and Derivatives (305)
	19	o__Lactobacillales (UID375)	177	350	163	76	207	64	3	0	0	73.49	20.72	8.22	P_bin19	15	0.007845	Mobile element protein	143	0.282051	Aerococcus viridans	Carbohydrates (133)
	20	k__Bacteria (UID203)	5449	104	58	56	18	16	5	9	0	24.03	14.02	24.71	P_bin20	5	0.021834	LSU ribosomal protein L14p (L23e)	20	0.4	Enhydrobacter	Fatty Acids, Lipids, and Isoprenoids (3)
	21	k__Bacteria (UID203)	5449	104	58	5	79	16	4	0	0	93.1	19.28	100	P_bin21	8	0.003521	Lipid A export ATP-binding/permease	98	0.207188	Macrococcus	Protein Metabolism (209)
	22	k__Bacteria (UID203)	5449	104	58	101	2	1	0	0	0	3.45	0.86	100	P_bin22	38	0.064189	Mobile element protein	39	0.171806	Exiguobacterium	DNA Metabolism (26)
	30	root (UID1)	5656	56	24	55	0	1	0	0	0	0.38	0.38	0	P_bin30	3	0.05	NADH-ubiquinone oxidoreductase	3	0.142857	Synechococcus sp. WH	
	33	root (UID1)	5656	56	24	54	2	0	0	0	0	8.33	0	0	P_bin33	5	0.017921	Mobile element protein	27	0.442623	Acinetobacter sp. ATCC	Membrane Transport (6)
	34	k__Bacteria (UID203)	5449	104	58	86	11	7	0	0	0	4.58	1.1	28.57	P_bin34	3	0.011628	Thymidylate synthase (EC 2.1.1.45)	9	0.088235	Arthrobacter arilaitensis	Phages, Prophages, Transposable
public houses (P_plus)	1	k__Bacteria (UID203)	5449	104	58	99	5	0	0	0	0	8.62	0	0	P_plus_bin1	7	0.008092	Mobile element protein	16	0.066946	Janibacter sp. HTCC2649	Carbohydrates (24)
	10	c__Bacilli (UID285)	586	325	181	18	270	37	0	0	0	93.09	12.57	94.59	P_plus_bin10	8	0.003754	Mobile element protein	89	0.19181	Macrococcus	Protein Metabolism (177)
	13	o__Actinomycetales (UID1530)	622	257	152	12	219	26	0	0	0	94.08	10.98	96.15	P_plus_bin13	21	0.005197	Mobile element protein	521	0.381406	Kocuria palustris PEL	Amino Acids and Derivatives (317)
	16	o__Actinomycetales (UID1530)	622	257	152	24	221	12	0	0	0	90.73	4.5	91.67	P_plus_bin16	33	0.008623	Mobile element protein	443	0.352426	Kocuria rhizophila	Amino Acids and Derivatives (312)
	21	f__Micrococcaceae (UID1623)	39	457	218	305	148	3	1	0	0	32.84	1.65	33.33	P_plus_bin21	18	0.010664	Mobile element protein	203	0.373162	Arthrobacter arilaitensis	Carbohydrates (77)
	23	k__Bacteria (UID203)	5449	104	58	94	10	0	0	0	0	7.84	0	0	P_plus_bin23	57	0.051677	Mobile element protein	42	0.124629	Kocuria rhizophila	Virulence, Disease and Defense (35)
	3	k__Bacteria (UID203)	5449	103	57	3	64	31	5	0	0	95.61	55.48	91.3	P_plus_bin3	36	0.005802	acetyltransferase, GNAT family	3810	0.846291	Exiguobacterium	Carbohydrates (529)
	4	k__Bacteria (UID203)	5449	104	58	99	3	2	0	0	0	0.78	0.31	0	P_plus_bin4	2	0.011765	Thymidylate synthase (EC 2.1.1.45)	4	0.047619	Pantoea ananatis LMG	Phages, Prophages, Transposable
	5	f__Enterobacteriaceae (UID5054)	223	876	305	111	457	276	30	2	0	90.68	37.88	45.77	P_plus_bin5	26	0.004153	Methyl-accepting chemotaxis protein I	206	0.175021	Pantoea ananatis LMG	Carbohydrates (502)
	6	o__Lactobacillales (UID355)	490	327	182	216	102	9	0	0	0	30.13	2.21	22.22	P_plus_bin6	10	0.01443	Mobile element protein	143	0.60084	Aerococcus viridans	Carbohydrates (29)
	8	k__Bacteria (UID203)	5449	104	58	53	29	18	4	0	0	37.4	12.85	76.67	P_plus_bin8	16	0.004799	Transcriptional regulator, LysR family	280	0.389972	Pantoea ananatis LMG	Carbohydrates (118)
	9	root (UID1)	5656	56	24	53	2	1	0	0	0	2.46	0.38	0	P_plus_bin9	26	0.05098	Mobile element protein	31	0.156566	Exiguobacterium	Phages, Prophages, Transposable
gowning area (UR)	0	f__Flavobacteriaceae (UID2817)	81	511	283	166	325	20	0	0	0	68.3	3.53	55	UR_bin0	12	0.004847	Two-component system response	16	0.035011	Flavobacterium	Amino Acids and Derivatives
	16	o__Pseudomonadales (UID4488)	185	813	308	465	273	64	9	2	0	44.24	11.59	38.83	UR_bin16	62	0.008713	Transcriptional regulator, LysR family	892	0.46775	Pseudomonas	Amino Acids and Derivatives
	17	o__Sphingomonadales (UID3310)	26	569	293	311	230	27	1	0	0	43.13	5.28	16.67	UR_bin17	73	0.016276	Mobile element protein	123	0.097156	Novosphingobium sp.	Carbonhydrates
	18	o__Sphingomonadales (UID3310)	26	569	293	37	497	35	0	0	0	92.7	9.33	85.71	UR_bin18	28	0.00546	Mobile element protein	320	0.255387	Sphingobium sp. AP49	Amino Acids and Derivatives (371)
	19	root (UID1)	5656	56	24	54	1	1	0	0	0	8.33	4.17	0	UR_bin19	231	0.053696	Mobile element protein	391	0.253238	Pseudomonas	Virulence, Disease and Defense (129)
	21	k__Bacteria (UID203)	5449	104	58	64	25	12	3	0	0	56.72	26.55	76.19	UR_bin21	66	0.005966	Transcriptional regulator, LysR family	2738	0.552796	Stenotrophomonas	Amino Acids and Derivatives
	24	o__Actinomycetales (UID1530)	622	259	152	34	191	31	3	0	0	85.8	13.18	92.5	UR_bin24	44	0.012185	conserved hypothetical protein	1290	0.746096	Propionibacterium	Carbonhydrates
	26	f__Rhizobiaceae (UID3564)	78	840	354	7	710	102	13	7	1	99.01	18.85	34.2	UR_bin26	242	0.022524	Mobile element protein	1846	0.456028	Agrobacterium	Carbohydrates (832)
	28	p__Bacteroidetes (UID2605)	350	316	210	232	83	1	0	0	0	25.05	0.48	100	UR_bin28	16	0.01435	Mobile element protein	58	0.203509	Sphingobacterium	Virulence, Disease and Defense (20)
	32	c__Alphaproteobacteria (UID3305)	564	349	230	32	307	10	0	0	0	88.12	3.41	50	UR_bin32	10	0.005008	diguanylate	83	0.148479	Micavibrio	Protein Metabolism (167)
	33	f__Moraxellaceae (UID4680)	86	689	365	40	506	130	11	2	0	92.75	24.59	84	UR_bin33	256	0.038694	Mobile element protein	79	0.089569	Acinetobacter sp. ATCC	Amino Acids and Derivatives (414)
	39	k__Bacteria (UID203)	5449	103	57	84	17	2	0	0	0	31.58	3.51	50	UR_bin39	94	0.014971	Glyoxylate reductase (EC 1.1.1.79) /	1497	0.650587	Pseudomonas	Amino Acids and Derivatives
	41	k__Bacteria (UID203)	5449	104	58	20	42	21	18	2	1	78.15	62.15	45.36	UR_bin41	33	0.004112	Inosine-uridine preferring nucleoside	1917	0.599437	Stenotrophomonas	Amino Acids and Derivatives
	7	k__Bacteria (UID203)	5449	104	58	39	52	10	3	0	0	52.32	7.21	10.53	UR_bin7	23	0.005852	Mobile element protein	1145	0.582994	Caulobacter crescentus	Membrane Transport (94)
	9	k__Bacteria (UID1453)	901	171	117	140	25	4	2	0	0	8.04	1.66	20	UR_bin9	22	0.043738	Mobile element protein	12	0.090909	Acinetobacter	Phages, Prophages, Transposable

Supplementary Table 3: Pan- and Core genome analysis of different built environments and species.

pan-genomes of different built environments

genome bin	CDS	CDS (without artefact fam.)	Pan CDS	Core CDS	Var CDS	Strain specific CDS	Core CDS (%)	Var CDS (%)	Strain spe. CDS (%)	Excluded CDS (%)
CR8 bin_21	4642	4642	4642	5	4637	4600	0.108	99.892	99.095	0
CR8 bin_33	5238	5238	5238	3	5235	5200	0.057	99.943	99.275	0
CR8 bin_8	3070	3070	3070	3	3067	3063	0.098	99.902	99.772	0
F bin_15	5169	5169	5169	4	5165	712	0.077	99.923	13.774	0
F bin_23	10627	10627	10627	7	10620	2551	0.066	99.934	24.005	0
F bin_31	7567	7567	7567	2	7565	1563	0.026	99.974	20.655	0
F bin_34	2183	2183	2183	2	2181	188	0.092	99.908	8.612	0
F bin_35	6345	6345	6345	5	6340	2926	0.079	99.921	46.115	0
F_plus bin_10	2695	2695	2695	2	2693	468	0.074	99.926	17.365	0
F_plus bin_13	4170	4170	4170	1	4169	1563	0.024	99.976	37.482	0
F_plus bin_14	2762	2762	2762	3	2759	289	0.109	99.891	10.463	0
F_plus bin_18	7064	7064	7064	3	7061	1381	0.042	99.958	19.55	0
F_plus bin_23	2222	2222	2222	3	2219	205	0.135	99.865	9.226	0
F_plus bin_30	6136	6136	6136	1	6135	6042	0.016	99.984	98.468	0
F_plus bin_4	9563	9563	9563	6	9557	6038	0.063	99.937	63.139	0
F_plus bin_9	8301	8301	8301	2	8299	1626	0.024	99.976	19.588	0
ICU bin_18	6265	6265	6265	8	6257	6167	0.128	99.872	98.436	0
ICU bin_24	3951	3951	3951	8	3943	3861	0.202	99.798	97.722	0
ICU bin_3	2826	2826	2826	6	2820	2816	0.212	99.788	99.646	0
L bin_0	3892	3892	3892	5	3887	711	0.128	99.872	18.268	0
L bin_10	2265	2265	2265	5	2260	156	0.221	99.779	6.887	0
L bin_17	5420	5419	5419	3	5416	1181	0.055	99.945	21.794	0
L_plus bin_11	2544	2544	2544	5	2539	144	0.197	99.803	5.66	0
L_plus bin_16	4973	4971	4971	4	4967	954	0.08	99.92	19.191	0
L_plus bin_6	3608	3608	3608	4	3604	523	0.111	99.889	14.496	0
P bin_10	3488	3488	3488	4	3484	901	0.115	99.885	25.831	0
P bin_14	6196	6196	6196	4	6192	1000	0.065	99.935	16.139	0
P bin_15	4987	4987	4987	6	4981	1473	0.12	99.88	29.537	0
P bin_18	2975	2975	2975	5	2970	709	0.168	99.832	23.832	0
P bin_21	2281	2281	2281	5	2276	202	0.219	99.781	8.856	0
P_plus bin_10	2109	2109	2109	4	2105	153	0.19	99.81	7.255	0
P_plus bin_13	3026	3026	3026	4	3022	755	0.132	99.868	24.95	0
P_plus bin_16	3130	3130	3130	4	3126	568	0.128	99.872	18.147	0
P_plus bin_3	6021	6021	6021	5	6016	864	0.083	99.917	14.35	0
P_plus bin_5	5086	5086	5086	6	5080	1626	0.118	99.882	31.97	0
UR bin_18	4216	4216	4216	0	4216	4096	0	100	97.154	0
UR bin_24	3332	3331	3331	0	3331	3325	0	100	99.82	0
UR bin_26	10242	10242	10242	0	10242	10061	0	100	98.233	0
UR bin_32	2545	2545	2545	0	2545	2504	0	100	98.389	0
UR bin_33	7639	7639	7639	0	7639	7504	0	100	98.233	0
UR bin_41	6116	6116	6116	0	6116	6037	0	100	98.708	0
UR bin_9	846	843	843	0	843	793	0	100	94.069	0

pan-genome of Acinetobacter

genome bin	CDS	CDS (without artefact fam.)	Pan CDS	Core CDS	Var CDS	Strain specific CDS	Core CDS (%)	Var CDS (%)	Strain spe. CDS (%)	Excluded CDS (%)
ICU bin_24	3951	3951	3951	1542	2409	784	39.028	60.972	19.843	0
CR8 bin_21	4642	4642	4642	1716	2926	366	36.967	63.033	7.885	0
F_plus bin_30	6136	6136	6136	2209	3927	3157	36.001	63.999	51.45	0
UR bin_33	7639	7639	7639	1802	5837	2857	23.589	76.411	37.4	0

pan-genome of confined built environments

genome bin	CDS	CDS (without artefact fam.)	Pan CDS	Core CDS	Var CDS	Strain specific CDS	Core CDS (%)	Var CDS (%)	Strain spe. CDS (%)	Excluded CDS (%)
CR8 bin_8	3070	3070	3070	0	3070	559	0	100	18.208	0
CR8 bin_21	4642	4642	4642	0	4642	410	0	100	8.832	0
CR8 bin_33	5238	5238	5238	0	5238	3575	0	100	68.251	0
UR bin_9	846	843	843	0	843	766	0	100	90.866	0
UR bin_18	4216	4216	4216	0	4216	4093	0	100	97.083	0
UR bin_24	3332	3331	3331	0	3331	970	0	100	29.12	0
UR bin_26	10242	10242	10242	0	10242	10001	0	100	97.647	0
UR bin_32	2545	2545	2545	0	2545	2503	0	100	98.35	0
UR bin_33	7639	7639	7639	0	7639	2988	0	100	39.115	0
UR bin_41	6116	6116	6116	0	6116	4189	0	100	68.492	0
ICU bin_3	2826	2826	2826	0	2826	478	0	100	16.914	0
ICU bin_18	6265	6265	6265	0	6265	5979	0	100	95.435	0
ICU bin_24	3951	3951	3951	0	3951	853	0	100	21.589	0

pan-genome of unrestricted built environments

genome bin	CDS	CDS (without artefact fam.)	Pan CDS	Core CDS	Var CDS	Strain specific CDS	Core CDS (%)	Var CDS (%)	Strain spe. CDS (%)	Excluded CDS (%)
F bin_15	5169	5169	5169	0	5169	712	0	100	13.774	0
F bin_23	10627	10627	10627	0	10627	2539	0	100	23.892	0
F bin_31	7567	7566	7566	0	7566	953	0	100	12.596	0
F bin_34	2183	2183	2183	0	2183	103	0	100	4.718	0
F bin_35	6345	6345	6345	0	6345	2921	0	100	46.036	0
F_plus bin_4	9563	9563	9563	0	9563	5943	0	100	62.146	0
F_plus bin_9	8301	8301	8301	0	8301	1620	0	100	19.516	0
F_plus bin_10	2695	2695	2695	0	2695	458	0	100	16.994	0
F_plus bin_13	4170	4170	4170	0	4170	1563	0	100	37.482	0
F_plus bin_14	2762	2762	2762	0	2762	289	0	100	10.463	0
F_plus bin_18	7064	7064	7064	0	7064	878	0	100	12.429	0
F_plus bin_23	2222	2222	2222	0	2222	111	0	100	4.995	0
F_plus bin_30	6136	6136	6136	0	6136	5965	0	100	97.213	0
P bin_10	3488	3488	3488	0	3488	896	0	100	25.688	0
P bin_14	6196	6195	6195	0	6195	549	0	100	8.862	0
P bin_15	4987	4987	4987	0	4987	1458	0	100	29.236	0
L_plus bin_6	3608	3608	3608	0	3608	514	0	100	14.246	0
L_plus bin_11	2544	2544	2544	0	2544	65	0	100	2.555	0
L_plus bin_16	4973	4971	4971	0	4971	437	0	100	8.791	0
P bin_18	2975	2975	2975	0	2975	708	0	100	23.798	0
P bin_21	2281	2281	2281	0	2281	54	0	100	2.367	0
P_plus bin_3	6021	6021	6021	0	6021	475	0	100	7.889	0
P_plus bin_5	5086	5086	5086	0	5086	1624	0	100	31.931	0
P_plus bin_10	2109	2109	2109	0	2109	69	0	100	3.272	0
P_plus bin_13	3026	3026	3026	0	3026	751	0	100	24.818	0
P_plus bin_16	3130	3130	3130	0	3130	561	0	100	17.923	0
L bin_0	3892	3892	3892	0	3892	680	0	100	17.472	0
L bin_10	2265	2265	2265	0	2265	69	0	100	3.046	0
L bin_17	5420	5419	5419	0	5419	598	0	100	11.035	0

Supplementary Table 4: A list of cleaning and disinfection reagents applied for various surfaces and purposes in the sampled built environments.

built environment											
public buildings (L)		public houses (P)		private houses (F)		intensive care unit (ICU)		gowning area (UR)		cleanroom (CR)	
floors	mechanically (broom)	floors	natural soaps	floors	all-purpose cleaners	hand desinfection	Desderman pure<br>Desmanol pure<br>Descoderm<br>Skinman Soft Protect<br>Sterilium classic pure<br>Sterillium LSG	surfaces	isopropanol 70%<br>JAMINAL PLUS<br>KLERCIDE-CR	surfaces	isopropanol 70%<br>JAMINAL PLUS<br>KLERCIDE-CR
								devices and products	isopropanol 70%	devices and products	isopropanol 70%<br>vapor phase H_2O_2<br>autoclaving
						skin antiseptic	Kodan forte (colored)<br>Kodan forte (colorless)<br>Betaseptic<br>Betaisodona standardized solution				
						mucosal antiseptic	Octenisept solution<br>Betaisodona standardized solution				
						surface desinfection	Incidin Plus				
						rapid desinfection (containing alcohol)	Incidin Liquid				
						rapid desinfection (nonalcoholic)	Acryl-des desinfection tissues<br>Acrylan				
						desinfectant for instruments (manually preparation)	Gigasept Instru AF<br>Sekusept plus<br>Sekusept active				

Supplementary Table 5: A list of cleaning and disinfection reagents including the exposure time applied for certain cases in the ICU at the state hospital in Graz, Austria.

disease / germ	range of application		
	hand desinfection	surface desinfection	
		rapid desinfection	routine desinfection
Norovirus	Desderman pure (30 sec)<br>Desmanol pure (30 sec)	Incidin liquid (10 min)	Incidin active 1% (1 hr)
Adenovirus	Desderman pure (30 sec)	Incidin liquid (10 min)	Incidin plus 1% (1 hr)
Rotavirus	Desderman pure (30 sec)<br>Desmanol pure (30 sec)<br>Descoderm (30 sec)	Incidin liquid (5 min)	Incidin plus 0.5% (1 hr)
Clostridium difficile	standard desinfectants according to cleaning and desinfection protocols	Incidin active 2% (15 min)	Incidin active 1% (30 min)
gas gangrene	standard desinfectants according to cleaning and desinfection protocols (in-depth cleaning)		
tuberculosis	standard desinfectants applied 2 times according to cleaning and desinfection protocols	Incidin liquid (5 min)	Incidin plus 0.5% (1 hr)
MRE (MRSA, ESBL etc.)<br>hepatitides, HIV<br>influenza A, B<br>measles, mumps<br>Meningococci<br>pertussis<br>Salmonellae<br>SARS<br>varicella	standard desinfectants according to cleaning and desinfection protocols		

Supplementary Table 6: Complete list of all primers used in the study.

primer name	target region	application	primer sequence
515f	V4 region of the 16S rRNA gene	16S amplicons	GTGCCAGCMGCCGCGGTAA
806r			GGACTACHVGGGTWTCTAAT
515F		qPCR	GTGCCAGCAGCCGC
927R			CCCGTCAATTYMTTTGAGTT

Supplementary Table 7: Summary on all quality reads of the shotgun metagenomics dataset.

samples	sequences (per each read)	sequence length	%GC	read	Phred Score (average quality per read; seq/q)
**CR8**	6.71E+07		44	1	>2.5E+07/37
				2	>2.0E+07/37
**UR**	5.35E+07		48	1	>1.8E+07/37
				2	>1.4E+07/37
**ICU**	3.30E+07		42	1	>1.6E+07/37
				2	>1.4E+07/37
**L**	3.03E+07		60	1	>8.0E+06/36
				2	>8.0E+06/36
**L_plus**	3.17E+07	50-150	62	1	>9.0E+06/36
				2	>8.0E+06/36
**P**	3.32E+07		60	1	>1.0E+07/36
				2	>7.0E+06/36
**P_plus**	3.28E+07		61	1	>9.0E+06/36
			60	2	>7.0E+06/36
**F**	3.97E+07		45	1	>1.4E+07/37
				2	>1.2E+07/37
**F_plus**	3.69E+07		46	1	>1.2E+07/37
				2	>1.2E+07/37

Supplementary Table 8: Summary of all contigs and scaffolds after assembly of the shotgun metagenomics dataset.

Contigs >= 100 nt	Number	Total length	Average	N50	Median	Largest
CR8	8603965	2010442497	233	251	173	88313
UR	6781605	1285900173	189	169	148	77951
ICU	5846714	1194191177	204	204	162	152957
L	3719862	616578790	165	144	140	112186
L_plus	3595693	606816993	168	146	141	192834
P	3609403	583352985	161	144	140	260735
P_plus	3529598	569566003	161	145	140	260738
F	3013516	528935438	175	143	138	128864
F_plus	3103321	529882363	170	142	138	98570

Contigs >= 500 nt	Number	Total length	Average	N50	Median	Largest
ICU	98994	73962331	747	625	574	152957
CR8	468389	338833151	723	667	613	88313
UR	103191	129616359	1256	1523	713	77951
L_plus	50525	64061830	1267	1449	771	192834
P	31304	47448008	1515	2299	766	260735
P_plus	29900	44378695	1484	2206	748	260738
F	59104	91192370	1542	2084	870	128864
F_plus	54071	82752023	1530	2032	867	98570
L	50240	60661672	1207	1324	750	112186

Scaffolds >= 100 nt	Number	Total length	Average	N50	Median	Largest
ICU	5846470	1194230266	204	204	162	152957
CR8	8602774	2010603824	233	251	173	88313
UR	6779560	1286163090	189	169	148	101382
L_plus	3594500	606967359	168	146	141	258085
P	3608497	583483719	161	144	140	260735
P_plus	3528733	569693266	161	145	140	260738
F	3011212	529221167	175	143	138	128864
F_plus	3101251	530124777	170	142	138	98570
L	3718903	616698401	165	144	140	155114

Scaffolds >= 500 nt	Number	Total length	Average	N50	Median	Largest
ICU	98764	74008162	749	626	574	152957
CR8	467352	339066479	725	668	612	88313
UR	101444	130012967	1281	1592	711	101382
L_plus	49546	64308268	1297	1520	772	258085
P	30472	47613011	1562	2510	763	260735
P_plus	29106	44538821	1530	2402	744	260738
F	57173	91638271	1602	2264	872	128864
F_plus	52403	83164916	1587	2194	871	98570
L	49474	60867124	1230	1371	751	155114

Supplementary Table 9: Settings for selected bioinformatic tools.

software tool	application	settings
Trimmomatic-0.32	data filtering	MIN_LENGTH=50, TAILCROP=0, HEADCROP=0, ILLUMINACLIP:TruSeq3-PE-2.fa:2:30:10:4:true, SLIDINGWINDOW: 5:20
MEGAN 5	single read visualization	files: paired_reads=yes, max_matches_per_read=100; GI Maps: gi_taxid-March2015X.bin, gi2kegg-Feb2015X.bin, gi2seed.map; LCA Params: min_score=50, max_expected=0.01, top_percent=10, min_support_percent=0, min_support=1, embed_in_main_file
Ray Meta-2.3.1	assembly	K=31, MINLEN=1000, MINCOV=5, READLEN=150
CONCOCT-0.4.0	binning	clusters=400, epsilon=1e-06, iterations=500, kmer_length=4, length_threshold=1000, pca_components=0.9, read_length=100, seed=1, total_percentage_pca=90
MaxBin-1.4.2	binning	min_contig_length=1000, marker genes 107,
Amphora2	taxonomic assignment	support_factor=(2/3.0), min_confidence=0.8, MIN_MARKERPERCENTAGE=0.2, NUM_BACT_MARKERS=31, NUM_ARCH_MARKERS=104
dRep-0.5.7	genome comparisons	MASH_sketch=1000, P_ani=0.9, S_algorithm='ANIn', S_ani=0.99, SkipMash=False, SkipSecondary=False, cent_index=None, clusterAlg='average', cov_thresh=0.1, coverage_method='larger', dry=False, n_PRESET='normal', operation='compare_wf', overwrite=False, processors=6, run_tax=False, warn_aln=0.25, warn_dist=0.25, warn_sim=0.98
iRep-1.1	replication rates of genomes	Line rate= 6, Lines per side= 1, Offset rate= 4, FTable chars= 10, Strings= unpacked, len divisor= 4, Difference-cover sample period= 1024, Endianness= little, Actual local endianness= little, Random seed= 0, Sizeofs= void*=8, int=4, long=8, size_t=8

Supplementary Table 10: Summary of applied statistics on the 16S rRNA gene amplicon dataset.

summary of applied statistics (16S rRNA gene amplicons)

Test	grouping category	metric	significance	details
MRPP	environmental confinement	weighted unifrac	0.001	A: 0.1618, delta 0.4224 of 0.504
	sampling location / surface		0.001	A: 0.1583, delta 0.4242 of 0.504
	environmental confinement	unweighted unifrac	0.001	A: 0.1392, delta 0.6759 of 0.7851
	sampling location		0.001	A: 0.1252, delta 0.6868 of 0.7851
adonis	environmental confinement	weighted unifrac	0.001	R2: 0.30668
	type of built environment		0.001	R2: 0.51265
	sampling location / surface		0.001	R2: 0.30079
	surface material		0.001	R2: 0.41641
ANOSIM	environmental confinement		0.001	R: 0.633738272592444
	type of built environment		0.001	R: 0.607909664422414
	sampling location / surface		0.001	R: 0.603552220205951
	surface material		0.001	R: 0.380902465808126
two-sided two sample Student's t-tests	environmental confinement		distances (maximum to minimum)	controlled vs. moderate controlled 0.6 > moderate controlled vs. uncontrolled 0.59 > controlled vs. Uncontrolled 0.4
	type of built environment			ICU_floor_vs._public_buildings_wp 0.65; public_house_vs._public_buildings_wp 0.36
	sampling location / surface			table_vs._floor 0.6; device_vs._table 0.37
	surface material			metal_polymer_vs._concrete_tiles 0.68; polymer_tiles_vs._stone 0.21
Mantel correlogram	humidity (H)			
	temperature (°C)			
	room volume (vol)			
	surface area (surf)			
	room area (room)			
	surface material (m)			
	geographic latitude (la)			
	geographic longitude (lo)			
	sea level (sl)			
	room height (h)			
BEST (BioEnv)	environmental variables	Bray-Curtis		
	sample		0.9773	
	sample,lo		0.9586	
	sample,la,lo		0.9705	
	sample,la,lo,sl		0.9425	
	sample,vol,la,lo,sl		0.9043	
	sample,vol,H,la,lo,sl		0.8846	
	sample,surf,vol,H,la,lo,sl		0.8659	
	sample,surf,h,vol,H,la,lo,sl		0.8355	
	sample,surf,room,h,vol,H,la,lo,sl		0.8044	
	sample,surf,room,h,vol,H,C,la,lo,sl		0.7738	
	sample,surf,room,h,vol,m,H,C,la,lo,sl		0.7518	

Supplementary Table 11: Summary of applied statistics on predicted functions from the 16S rRNA gene amplicon dataset with PICRUSt.

summary of applied statistics (PICRUSt predictions of 16S rRNA gene amplicons)

Test	grouping category	metric	significance	details
MRPP	environmental confinement	Bray-Curtis	0.001	A: 0.181, delta 0.06969 of 0.08509
	type of built environment		0.001	A: 0.3074, delta 0.05893 of 0.08509
	sampling location / surface		0.001	A: 0.2373, delta 0.0649 of 0.08509
	surface material		0.001	A: 0.2896, delta 0.06045 of 0.08509
adonis	environmental confinement		0.001	R2: 0.32734
	type of built environment		0.001	R2: 0.55937
	sampling location / surface		0.001	R2: 0.43945
	surface material		0.001	R2: 0.51541
ANOSIM	environmental confinement		0.001	R: 0.482880658436213
	type of built environment		0.001	R: 0.508214886053013
	sampling location / surface		0.001	R: 0.740354908750047
	surface material		0.001	R: 0.395628422683383
two-sided two sample Student's t-tests	environmental confinement		distances (maximum to minimum)	moderate controlled vs. uncontrolled 0.1 > moderate controlled vs. controlled 0.1 > controlled vs. uncontrolled 0.06
	type of built environment			ICU_workplace_vs._private_house 0.14; gowning_area_vs._public_house 0.04
	sampling location / surface			table_vs._floor 0.12; device_vs._table 0.06
	surface material			polymer_tiles_vs._furnished_wood 0.15; polymer_tiles_vs._stone 0.04
Mantel correlogram	room volume (vol)			
	humidity (H)			
	temperature (°C)			
	room area (room)			
	surface area (surf)			
	surface material (m)			
	geographic latitude (la)			
	geographic longitude (lo)			
	sea level (sl)			
	room height (h)			
BEST (BioEnv)	environmental variables			
	sample		0.8918	
	sample, vol		0.8996	
	sample, h, vol		0.8847	
	sample, surf, h, vol		0.8645	
	sample, h, vol, la, lo		0.8448	
	sample, surf, h, vol, la, lo		0.835	
	sample, surf, h, vol, la, lo, sl		0.8123	
	sample, surf, h, vol, H, la, lo, sl		0.7924	
	sample, surf, room, h, vol, H, la, lo, sl		0.7543	
	sample, surf, room, h, vol, m, H, la, lo, sl		0.7262	
	sample, surf, room, h, vol, m, H, C, la, lo, s		0.7024	

Supplementary Table 12: Read statistics of the 16S rRNA gene amplicon dataset.

**16S rRNA gene amplicons**	
Number of samples	61
Number of OTUs (operational taxonomic units)	10814
Number of sequences	837216

Number of sequences per sample	
L5	225
L1.PMA	254
ICU.f.1	2052
ICU.f.5	2888
CR.7a	2928
ICU.f.4	4424
L8.PMA	4576
F2.PMA	5227
ICU.d.6	5308
F2	6444
ICU.d.11	6635
ICU.w.23	6778
ICU.w.18	7057
ICU.f.2	7168
UR.7	7255
L3.PMA	7307
L4.PMA	7543
ICU.w.17	7721
ICU.d.15	7841
P3	7920
L5.PMA	7965
ICU.d.9	8088
ICU.w.22	8221
ICU.d.13	8446
F3	8616
ICU.d.7	9243
ICU.w.24	9627
ICU.d.8	9628

Number of sequences per sample	
ICU.d.14	10228
ICU.d.10	10305
ICU.w.19	10504
F3.PMA	10846
ICU.f.3	10877
L1	10943
ICU.d.16	11185
ICU.d.12	11408
L6.PMA	12149
ICU.w.21	13147
P3.PMA	13454
ICU.w.20	14835
L3	14896
L9	15899
L7.PMA	16261
P1.PMA	18562
CR.7b	18888
P2.PMA	19242
L7	19359
L6	21036
P1	21321
L4	24486
F1	25455
F1.PMA	25670
L2	26425
UR	26913
P2	27974
L8	28344
F0	28566
L9.PMA	30456
F0.PMA	35436
CR.8	36930
L2.PMA	37831

summary of reads	
Minimum	225
Maximum	37831
Median	10504
Mean	13724.852
Std. dev.	9289.725

Supplementary References

1. Oberauner, L. *et al.* The ignored diversity: complex bacterial communities in intensive care units revealed by 16S pyrosequencing. *Sci. Rep.* **3,** 1413 (2013).
2. Koskinen, K. *et al.* Microbial biodiversity assessment of the European Space Agency's ExoMars 2016 mission. *Microbiome* **5,** 143 (2017).
3. Sinha, R. *et al.* Assessment of variation in microbial community amplicon sequencing by the Microbiome Quality Control (MBQC) project consortium. *Nat. Biotechnol.* **35,** 1077–1086 (2017).
4. Adams, R. I., Bateman, A. C., Bik, H. M. & Meadow, J. F. Microbiota of the indoor environment: a meta-analysis. *Microbiome* **3,** 49 (2015).
5. Chase, J. *et al.* Geography and Location Are the Primary Drivers of Office Microbiome Composition. *mSystems* **1,** 1–18 (2016).
6. Nicholson, W. L., Schuerger, A. C. & Race, M. S. Migrating microbes and planetary protection. *Trends Microbiol.* **17,** 389–392 (2009).
7. Rummel, J. D., Stabekis, P. D., Devincenzi, D. L. & Barengoltz, J. B. COSPAR'S planetary protection policy: A consolidated draft. *Adv. Sp. Res.* **30,** 1567–1571 (2002).
8. Rozov, R. *et al.* Recycler: an algorithm for detecting plasmids from *de novo* assembly graphs. *Bioinformatics* btw651 (2016). doi:10.1093/bioinformatics/btw651
9. Bankevich, A. *et al.* SPAdes: A New Genome Assembly Algorithm and Its Applications to Single-Cell Sequencing. *J. Comput. Biol.* **19,** 455–477 (2012).
10. Li, H. & Durbin, R. Fast and accurate long-read alignment with Burrows-Wheeler transform. *Bioinformatics* **26,** 589–95 (2010).
11. Zhu, W., Lomsadze, A. & Borodovsky, M. Ab initio gene identification in metagenomic sequences. *Nucleic Acids Res.* **38,** 1–15 (2010).
12. Camacho, C. *et al.* BLAST+: Architecture and applications. *BMC Bioinformatics* **10,** 421 (2009).
13. Kanehisa, M. & Goto, S. KEGG: Kyoto encyclopedia of genes and genomes. *Nucleic Acids Res.* **28,** 27–30 (2000).
14. Bairoch, A. *et al.* The Universal Protein Resource (UniProt). *Nucleic Acids Res.* **33,** 154–159 (2005).

15. Jia, B. *et al.* CARD 2017: Expansion and model-centric curation of the comprehensive antibiotic resistance database. *Nucleic Acids Res.* **45,** D566–D573 (2017).

16. Vallenet, D. *et al.* MaGe: a microbial genome annotation system supported by synteny results. *Nucleic Acids Res.* **34,** 53–65 (2006).

17. Chen, L., Zheng, D., Liu, B., Yang, J. & Jin, Q. VFDB 2016: Hierarchical and refined dataset for big data analysis - 10 years on. *Nucleic Acids Res.* **44,** D694–D697 (2016).

18. Joensen, K. G. *et al.* Real-time whole-genome sequencing for routine typing, surveillance, and outbreak detection of verotoxigenic Escherichia coli. *J. Clin. Microbiol.* **52,** 1501–1510 (2014).

19. Miele, V., Penel, S. & Duret, L. Ultra-fast sequence clustering from similarity networks with SiLiX. *BMC Bioinformatics* **12,** 116 (2011).

20. Cury, J., Jové, T., Touchon, M., Néron, B. & Rocha, E. P. Identification and analysis of integrons and cassette arrays in bacterial genomes. *Nucleic Acids Res.* **44,** 4539–4550 (2016).

21. Vernikos, G. S. & Parkhill, J. Interpolated variable order motifs for identification of horizontally acquired DNA: Revisiting the Salmonella pathogenicity islands. *Bioinformatics* **22,** 2196–2203 (2006).

22. Waack, S. *et al.* Score-based prediction of genomic islands in prokaryotic genomes using hidden Markov models. *BMC Bioinformatics* **7,** 1–12 (2006).

23. Caporaso, J. G. *et al.* QIIME allows analysis of high- throughput community sequencing data. *Nat. Methods* **7,** 335–336 (2010).

24. Bolyen, E. *et al.* QIIME 2: Reproducible, interactive, scalable, and extensible microbiome data science. *PeerJ Prepr.* (2018). doi:10.7287/peerj.preprints.27295v1

25. Schwendner, P. *et al.* Preparing for the manned Mars journey: Microbiome dynamics in the confined Mars500 habitat during simulated Mars flight and landing. *Microbiome, Submitt.* **5,** 129 (2017).

26. Mahnert, A., Haratani, M., Schmuck, M. & Berg, G. Enriching beneficial microbial diversity of indoor plants and their surrounding built environment with biostimulants. *Front. Microbiol.* (2018). doi:10.3389/fmicb.2018.02985

27. Schloss, P. D. *et al.* Introducing mothur: Open-source, platform-independent, community-supported software for describing and comparing microbial communities. *Appl. Environ. Microbiol.* **75,** 7537–7541 (2009).

28. Edgar, R. C. Search and clustering orders of magnitude faster than BLAST. *Bioinformatics* **26,** 2460–1 (2010).

29. Langille, M. G. I. *et al.* Predictive functional profiling of microbial communities using 16S rRNA marker gene sequences. *Nat. Biotechnol.* **31,** 814–21 (2013).

30. Ward, T. *et al.* BugBase Predicts Organism Level Microbiome Phenotypes. *bioRxiv* 1–19 (2017). doi:10.1101/133462

31. Shannon, P. *et al.* Cytoscape: a software environment for integrated models of biomolecular interaction networks. *Genome Res.* **13,** 2498–504 (2003).

32. Moissl-Eichinger, C. *et al.* Quo vadis? Microbial profiling revealed strong effects of cleanroom maintenance and routes of contamination in indoor environments. *Sci. Rep.* **5,** 9156 (2015).

33. Bokulich, N. A. *et al.* q2-sample-classifier: machine-learning tools for microbiome classification and regression. *bioRxiv* (2018). doi:10.1101/306167

34. Clarke, K. R. & Ainsworth, M. A method of linking multivariate community structure to environmental variables. *Mar. Ecol. Prog. Ser.* **92,** 205–219 (1993).

35. Morgan, X. C. *et al.* Dysfunction of the intestinal microbiome in inflammatory bowel disease and treatment. *Genome Biol.* **13,** R79 (2012).

36. Morton, J. T. *et al.* Balance Trees Reveal Microbial Niche Differentiation. *mSystems* **2,** 1–11 (2017).

37. Bokulich, N. A. *et al.* q2-longitudinal : Longitudinal and Paired-Sample Analyses of Microbiome Data. *mSystems* **3,** 1–9 (2018).
