# Supplementary material for: Man-made microbial resistances in built environments
Source: Nat Commun. 2019 Feb 27;10:968. doi: 10.1038/s41467-019-08864-0 (PMC6393488; doi:10.1038/s41467-019-08864-0)
Supplement: Supplementary file 3 — Reporting Summary [file 41467_2019_8864_MOESM3_ESM.pdf]

nature research

Corresponding author(s): Alexander Mahnert

Reporting Summary

Nature Research wishes to improve the reproducibility of the work that we publish. This form provides structure for consistency and transparency in reporting. For further information on Nature Research policies, see Authors & Referees and the Editorial Policy Checklist.

Statistical parameters

When statistical analyses are reported, confirm that the following items are present in the relevant location (e.g. figure legend, table legend, main text, or Methods section).

n/a	Confirmed	
☐	☒	The exact sample size (n) for each experimental group/condition, given as a discrete number and unit of measurement
☐	☒	An indication of whether measurements were taken from distinct samples or whether the same sample was measured repeatedly
☐	☒	The statistical test(s) used AND whether they are one- or two-sided<br>*Only common tests should be described solely by name; describe more complex techniques in the Methods section.*
☐	☒	A description of all covariates tested
☐	☒	A description of any assumptions or corrections, such as tests of normality and adjustment for multiple comparisons
☐	☒	A full description of the statistics including central tendency (e.g. means) or other basic estimates (e.g. regression coefficient) AND variation (e.g. standard deviation) or associated estimates of uncertainty (e.g. confidence intervals)
☐	☒	For null hypothesis testing, the test statistic (e.g. F, t, r) with confidence intervals, effect sizes, degrees of freedom and P value noted<br>*Give P values as exact values whenever suitable.*
☒	☐	For Bayesian analysis, information on the choice of priors and Markov chain Monte Carlo settings
☒	☐	For hierarchical and complex designs, identification of the appropriate level for tests and full reporting of outcomes
☒	☐	Estimates of effect sizes (e.g. Cohen's d, Pearson's r), indicating how they were calculated
☒	☐	Clearly defined error bars<br>*State explicitly what error bars represent (e.g. SD, SE, CI)*

Our web collection on statistics for biologists may be useful.

Software and code

Policy information about availability of computer code

Data collection	Rotor-Gene™ 6000 real – time rotary analyzer (Corbett Research, Sydney, Australia)<br>Illumina MiSeq v3 and HiSeq 2500 Control Software (Eurofins Genomics GmbH, Ebersberg, Germany)<br>Roche GS FLX+ 454 pyrosequencer Analyzer Software (GATC Biotech, Konstanz, Germany)
Data analysis	Trimmomatic-0.32, MEGAN 5, Ray Meta-2.3.1, CONCOCT-0.4.0, MaxBin-1.4.2, Amphora2, dRep-0.5.7, iRep-1.1, CheckM, RAST 2.0, MaGe v3.11.1, IMG, KEGG FTP release 2017-03-27 database, KEGG Feb2015 gi map, Uniref90 release 2016_10 database, CARD 1.1 database, SEED Feb2015 gi map, NCBI nr taxid March2015 gi map, Recycler, PICA, SPAdes 3, Burrows-Wheeler aligner, Metagenemark, VFDB database, VirulenceFinder 2.0, SiLiX , IntegronFinder 1.5.2, RGP Finder, AlienHunter, SIGI-HMM, , QIIME 1.9.1 , QIIME 2 (versions 2017.10. to 2018.11), Mothur 1.36.0, FASTX-toolkit 0.0.13, USEARCH 6, Silva database 119, Greengenes 13_8 release, PICRUSt, BugBase, Cytoscape 2.8.1. , MaAsLin 2018-09-07, R 3.5.1, MS Office, InkScape 0.92.3, GIMP 2.10.6

For manuscripts utilizing custom algorithms or software that are central to the research but not yet described in published literature, software must be made available to editors/reviewers upon request. We strongly encourage code deposition in a community repository (e.g. GitHub). See the Nature Research guidelines for submitting code & software for further information.

Data

Policy information about availability of data

All manuscripts must include a data availability statement. This statement should provide the following information, where applicable:

- Accession codes, unique identifiers, or web links for publicly available datasets
- A list of figures that have associated raw data
- A description of any restrictions on data availability

All raw data was deposited in the European Nucleotide Archive under project PRJEB27640. Processed shotgun reads are accessible in MG-RAST (http://metagenomics.anl.gov/) under projects 9258 and 10962, binned contigs and scaffolds are deposited in IMG/M (https://img.jgi.doe.gov/) through the Genome Online Database GOLD (https://gold.jgi.doe.gov/) and in MicroScope – the Microbial Genome Annotation & Analysis Platform (http://www.genoscope.cns.fr/agc/microscope/home/) and are available on request. Processed 16S rRNA gene amplicon reads were deposited in Qiita (see Ref. 57, https://qiita.ucsd.edu/) under study 10071. 16S rRNA gene amplicons from the ICU were published and deposited before (see Ref. 33).

Field-specific reporting

Please select the best fit for your research. If you are not sure, read the appropriate sections before making your selection.

☒ Life sciences ☐ Behavioural & social sciences ☐ Ecological, evolutionary & environmental sciences

For a reference copy of the document with all sections, see nature.com/authors/policies/ReportingSummary-flat.pdf

Life sciences study design

All studies must disclose on these points even when the disclosure is negative.

Sample size	Sample size calculations were not performed due to the retrorespective manner of this study.
Data exclusions	No data was excluded.
Replication	Beside technical replicates, samples were grouped into respective categories (ICU, cleanroom, gowning area, public buildings, public houses, private houses) for biological replicates.
Randomization	Not applicable. This was not an interventional study.
Blinding	Not applicable. This was not an interventional study.

Reporting for specific materials, systems and methods

Materials & experimental systems

n/a	Involved in the study	
☒	☐	Unique biological materials
☒	☐	Antibodies
☒	☐	Eukaryotic cell lines
☒	☐	Palaeontology
☒	☐	Animals and other organisms
☒	☐	Human research participants

Methods

n/a	Involved in the study	
☒	☐	ChIP-seq
☒	☐	Flow cytometry
☒	☐	MRI-based neuroimaging
